# Oncogenic Roles of Laminin Subunit Gamma‐2 in Intrahepatic Cholangiocarcinoma via Promoting EGFR Translation

**DOI:** 10.1002/advs.202309010

**Published:** 2024-03-25

**Authors:** Jianjuan Zhang, Fubo Ji, Yaqi Tan, Lei Zhao, Yongzhi Zhao, Jiaxin Liu, Liyuan Shao, Jiong Shi, Meihua Ye, Xianglei He, Jianping Jin, Bin Zhao, Jun Huang, Stephanie Roessler, Xin Zheng, Junfang Ji

**Affiliations:** ^1^ The MOE Key Laboratory of Biosystems Homeostasis & Protection Zhejiang Provincial Key Laboratory for Cancer Molecular Cell Biology Life Sciences Institute Zhejiang University Hangzhou Zhejiang 310058 China; ^2^ Center for Life Sciences Shaoxing Institute Zhejiang University Shaoxing Zhejiang 321000 China; ^3^ Cancer Center Zhejiang University Hangzhou Zhejiang 310058 China; ^4^ Shandong Cancer Hospital and Institute Shandong Cancer Hospital of Shandong First Medical University Jinan Shandong Province 250117 China; ^5^ Department of Pathology Nanjing Drum Tower Hospital The Affiliated Hospital of Nanjing University Medical School Nanjing Jiangsu Province 210008 China; ^6^ Zhejiang Provincial People's Hospital Hangzhou Zhejiang 310014 China; ^7^ Institute of Pathology University Hospital Heidelberg 69120 Heidelberg Germany; ^8^ Taoharmony Biotech L.L.C. Hangzhou Zhejiang 310018 China

**Keywords:** EGFR, glycosylation, intrahepatic cholangiocarcinoma, *LAMC2*, protein translation

## Abstract

Intrahepatic cholangiocarcinoma (iCCA) is a highly lethal biliary epithelial cancer in the liver. Here, Laminin subunit gamma‐2 (*LAMC2*) with important oncogenic roles in iCCA is discovered. In a total of 231 cholangiocarcinoma patients (82% of iCCA patients) across four independent cohorts, *LAMC2* is significantly more abundant in iCCA tumor tissue compared to normal bile duct and non‐tumor liver. Among 26.3% of iCCA patients, *LAMC2* gene is amplified, contributing to its over‐expression. Functionally, silencing *LAMC2* significantly blocks tumor formation in orthotopic iCCA mouse models. Mechanistically, it promotes EGFR protein translation via interacting with nascent unglycosylated EGFR in the endoplasmic reticulum (ER), resulting in activated EGFR signaling. *LAMC2*‐mediated EGFR translation also depends on its interaction with the ER chaperone BiP via their C‐terminus. Together *LAMC2* and BiP generate a binding “pocket” of nascent EGFR and facilitate EGFR translation. Consistently, *LAMC2*‐high iCCA patients have poor prognosis in two iCCA cohorts. *LAMC2*‐high iCCA cells are highly sensitive to EGFR tyrosine kinase inhibitors (TKIs) treatment both in vitro and in vivo. Together, these data demonstrate *LAMC2* as an oncogenic player in iCCA by promoting EGFR translation and an indicator to identify iCCA patients who may benefit from available EGFR‐targeted TKIs therapies.

## Graphical abstract

In intrahepatic cholangiocarcinoma (iCCA), *LAMC2* gene is amplified, leading to the increased levels of *LAMC2* protein. Within the endoplasmic reticulum, the increased *LAMC2* together with BiP interacted with newly synthesized EGFR, promoting EGFR protein translation. Consequently, the *LAMC2*/EGFR axis contributed to iCCA carcinogenesis. iCCA tumors with high levels of *LAMC2* expression exhibited sensitivity to EGFR tyrosine kinase inhibitors.

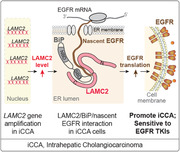

## 1 Introduction

Intrahepatic cholangiocarcinoma (iCCA) is one of the most lethal malignancies, with an overall 5‐year survival rate of 5–10%. As the second most common primary liver cancer, its incidence has been continuously increasing in recent years.^[^ ^1^, ^2^, ^3^ ^]^ Liver resection and liver transplantation are potentially curative treatment options for very early stage iCCA. However, the majority of iCCAs are often diagnosed at an unresectable stage. As for patients with advanced‐stage or unresectable iCCA tumors, the available standard systemic chemotherapy (gemcitabine and cisplatin) provides only minimal benefits.^[^ ^1^ ^]^ Most recently, two types of targeted therapeutic agents have been approved as second‐line treatment for patients harboring key iCCA oncogenic genomic alterations, i.e., *IDH1* mutation and *FGFR* fusion. They are Ivosidenib (mutant IDH1 inhibitor) as well as pemigatinib and infigratinib (FGFR fusion inhibitors), showing encouraging improvement in increasing patients’ survival rates.^[^ ^4^, ^5^ ^]^ However, *IDH1* mutation and *FGFR* fusion only occur in a limited iCCA population and the resistance typically develops within months.^[^ ^6^ ^]^ In this case, more efforts are needed to continue investigating the key oncogenic events of iCCA and to explore their potential therapeutic applications.

EGFR has been recognized as an effective therapeutic target in various cancer types with approved EGFR tyrosine kinase inhibitors (TKIs) for clinical use. However, *EGFR* amplifications and mutations occurred in less than 5% of iCCA cases. Nonetheless, over‐expression of EGFR exhibited in 10–44% of iCCA patients.^[^ ^7^, ^8^ ^]^ Additionally, a subtype of iCCA patients was previously reported with active EGFR signaling.^[^ ^9^ ^]^ Clinical trials have been conducted to evaluate the efficacy of EGFR TKIs and neutralizing antibodies in iCCA patients, although the focus has primarily been on combining these treatments with chemotherapy drugs rather than using EGFR TKIs alone. Unfortunately, early phase clinical trials ended with negative results of EGFR TKIs in iCCA patients. The EGFR TKI erlotinib and EGFR neutralizing antibodies (cetuximab and panitumumab) all showed only minimal or nearly no significant improvement in patient survival when combining them respectively with chemotherapy compared to chemotherapy alone.^[^ ^10^, ^11^, ^12^ ^]^ Till now, it remains unclear whether certain molecular subtypes of iCCA patients might benefit from clinical EGFR‐targeted therapy.

We aimed to identify key oncogenic events specific to iCCA and to explore the potential therapeutic strategies for this condition. In this study, our findings revealed *LAMC2* as a key oncogenic molecule in iCCA patients via *LAMC2*/BiP/EGFR axis and as a potential indicator in suggesting iCCA patients for available EGFR TKIs therapies.

## 2 Results

### 2.1 *LAMC2* Exhibited a Specific High Expression Level in iCCA Tumor Tissues

**Figure 1:**
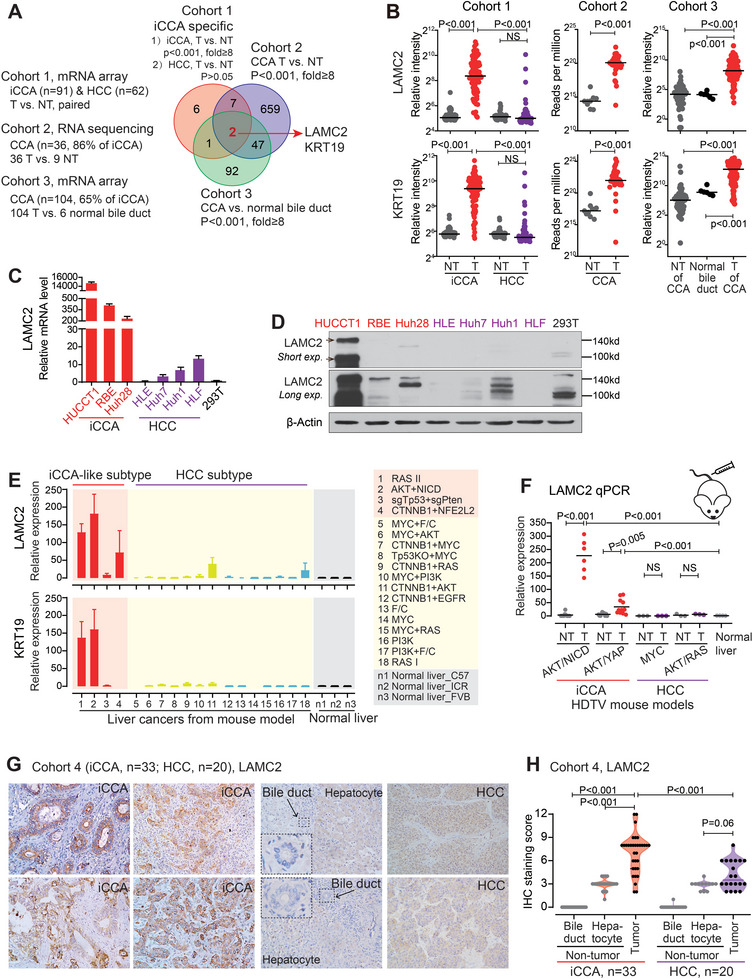
*LAMC2* expressed a specific high level in iCCA tumors. A) Screening of iCCA‐specific genes in Cohorts 1–3. B) *LAMC2* and KRT19 expression levels in Cohorts 1–3. C) *LAMC2* mRNA expression was examined by qRT‐PCR. D) *LAMC2* protein level was examined by Western blot. E) *LAMC2* and KRT19 mRNA levels in tumors from 18 liver cancer mouse models and in normal mouse livers. F) qRT‐PCR examination of *LAMC2* mRNA level in tumor and non‐tumors from various liver cancer mouse models. G) Representative images of *LAMC2* IHC staining in Cohort 4. H) *LAMC2* IHC staining score in tumors, bile duct cells, and hepatocytes from iCCA and HCC patients. B,F,H) Student's *t*‐test was used. NS, not significant. T, tumor; NT, non‐tumor.

Liver, the anatomical location of iCCA, consists primarily of hepatocytes with a minority of cholangiocytes and is also the site of hepatocellular carcinoma (HCC, the most common primary liver cancer). To globally identify iCCA‐specific oncogenic genes, we thus integrated transcriptome data from three clinical cohorts, including tissues from normal bile ducts as well as tumor and non‐tumor liver tissues from cholangiocarcinoma patients (iCCA, 82%) and HCC patients (**Figure** **1**A). Principle component analysis revealed that non‐tumor liver tissues from iCCA and HCC patients were tightly clustered together while their tumor tissues were two distinct groups (iCCA and HCC groups) (Figure S1A, Supporting Information, Cohort 1), highlighting the necessity of including HCC and bile ducts as controls. Through class comparisons in Cohorts 1–3, Laminin Subunit Gamma 2 (*LAMC2*) and KRT19 were identified as key candidates upon the stringent criteria, i.e., 8 times higher expression level in CCA tumor versus non‐tumor liver tissues (*p* < 0.001) and normal bile duct cells (*p* < 0.001), but no difference in HCC tumor versus non‐tumor liver tissues (*p* > 0.05) (Figure 1A,B). KRT19 is a well‐known biliary differentiation marker and presents a high expression level in iCCA.^[^ ^13^ ^]^ For *LAMC2*, a few articles reported that it was up‐regulated in CCA, contributed to invasive features of CCA cells, and was related to poor prognosis of patients.^[^ ^14^ ^]^ Therefore, *LAMC2* was chosen for further exploration since its function remained largely unknown in iCCA carcinogenesis.

The specific high expression of *LAMC2* was noticed in three iCCA cell lines in comparison with four HCC cell lines and 293T cells, at both the mRNA (Figure 1C) and protein levels (Figure 1D). Single‐cell RNA‐sequencing data of human liver cancers, including HCC and iCCA,^[^ ^15^ ^]^ portrayed a noticeably higher expression of *LAMC2* in iCCA tumor cells compared to other pathological types of liver cancer cells and tumor immune microenvironment cells (Figure S1B, Supporting Information). Furthermore, from hydrodynamic tail vein injection (HDTV) orthotopic liver cancer mouse models driven by various oncogenes,^[^ ^16^ ^]^ RNA sequencing data showed that *LAMC2* exhibited a significantly higher level in tumors from the iCCA‐like tumor subtype than HCC subtype and normal livers (Figure 1E). Such an iCCA‐specific expression panel of *LAMC2* was similar to KRT19, if not even better. Comparable data were also obtained via the qRT‐PCR method in mouse orthotopic liver cancers (Figure 1F).

Immunohistochemistry (IHC) staining of *LAMC2* was then performed in FFPE tissues in Cohort 4, which included 33 iCCA patients and 20 HCC patients (Table S2, Supporting Information). As shown in Figure 1G, *LAMC2* IHC staining was very strong in most iCCA tumor tissues, but no staining in normal bile duct cells or other non‐tumor environment cells. Furthermore, only faint *LAMC2* staining was noticed in hepatocytes, while weak and medium *LAMC2* staining was observed in HCC tumor tissues. Quantitative data consistently showed that *LAMC2* staining was significantly higher in iCCA tumor tissues versus bile duct cells, hepatocytes, and HCC tumors, respectively (*p* < 0.001 for each comparison, Figure 1H). Taken together, these data demonstrated a specific high expression of *LAMC2* in iCCA tumor tissues within the liver.

### 2.2 *LAMC2* Gene Amplification Contributed to the High Expression of *LAMC2* in iCCA

**Figure 2:**
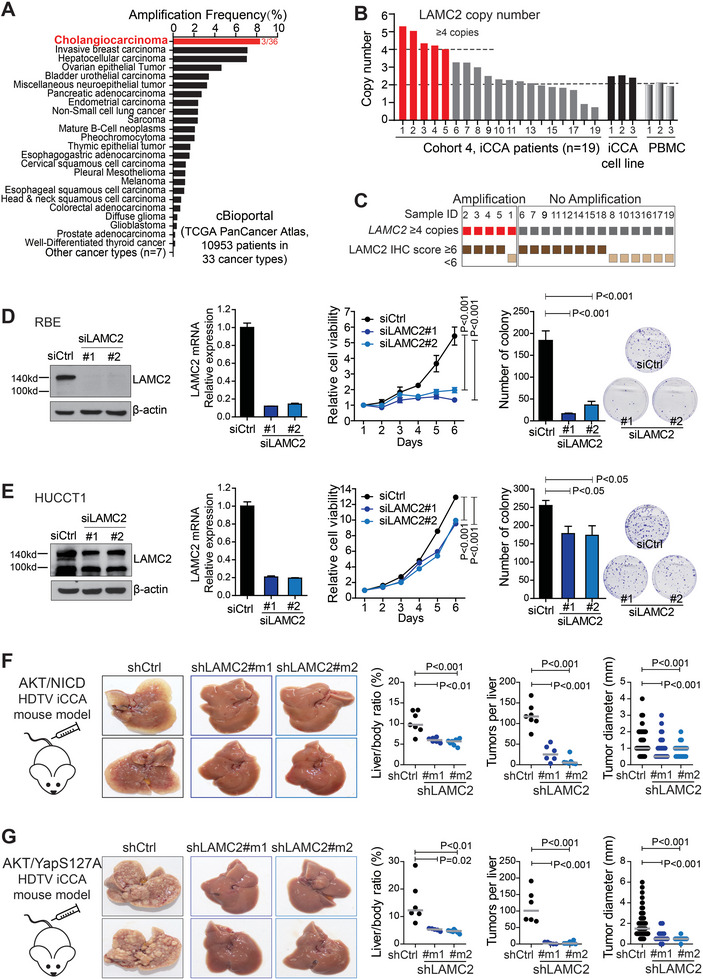
*LAMC2* was amplified in iCCA and silencing *LAMC2* inhibited iCCA both in vitro and in vivo. A) *LAMC2* amplification frequency of 10 953 patients from 33 cancer types in cBioPortal database. B) *LAMC2* copy number detection in iCCA tumors from Cohort 4 (*n* = 19), 3 iCCA cell lines (HUCCT1, RBE, Huh28) and PBMCs. C) *LAMC2* IHC staining score in iCCA patients with or without *LAMC2* copy number ≥ 4 from Cohort 4. D) Cell viability and colony formation in RBE cells transfected with control or *LAMC2* siRNA. E) Cell viability and colony formation in HUCCT1 cells transfected with control or *LAMC2* siRNA. F) iCCA formation in AKT/NICD‐induced iCCA mouse model with or without silencing *LAMC2* by shRNAs. G) iCCA formation in AKT/YapS127A‐induced iCCA mouse model with or without silencing *LAMC2* by shRNAs. D,E) Two‐way ANOVA was used for cell viability assay. Student's *t*‐test was used for colony formation assay. F,G) Student's *t*‐test was used. PBMC, peripheral blood mononuclear cell.

The genetic alteration of *LAMC2* gene was investigated. Analysis was performed with genetic data from cBioportal, including a total of 10 953 patients from 33 different cancer types (TCGA PanCancer Atlas Studies). It revealed *LAMC2* gene amplification in various cancer patients among all 33 cancer types, with the highest amplification frequency (8.3%) residing in CCA patients (**Figure** **2**A). No *LAMC2* mutations were noticed in CCA.

The *LAMC2* amplification in iCCA was then validated in Cohort 4. Genomic DNA was extracted from FFPE tissues of 19 iCCA tumors in Cohort 4 and gene copy number assay was performed with a copy number ≥4 as DNA amplification. In this cohort, five out of nineteen iCCA tumors (26.3%) presented *LAMC2* copy number ≥4, indicating a noticeable *LAMC2* amplification in iCCAs (Figure 2B). Furthermore, iCCA tumors with *LAMC2* amplification exhibited strong *LAMC2* IHC staining (IHC score ≥6). As shown in Figure 2C, 80% of iCCAs with *LAMC2* amplification (4 out of 5) had strong *LAMC2* IHC staining, while 57.1% of iCCAs without *LAMC2* amplification (8 out of 14) exhibited strong *LAMC2* staining. Thus, *LAMC2* gene amplification occurred in iCCA, which partially contributed to the high expression of *LAMC2* in iCCA tumors.

### 2.3 Silencing *LAMC2* Inhibited iCCA Malignancy Features In Vitro and Blocked Orthotopic iCCA Formation In Vivo

In iCCA cell lines, the silencing of *LAMC2* using siRNA significantly inhibited cell proliferation and colony formation of iCCA cells (Figure 2D,E). Similar results were acquired when using *LAMC2* shRNA in these iCCA cells (Figure S2A–D, Supporting Information). Moreover, *LAMC2* knockdown also reduced cell migration (Figure S2E,F, Supporting Information), as reported in extrahepatic CCA.^[^ ^17^ ^]^

In two HDTV orthotopic iCCA mouse models driven by AKT/NICD and AKT/YapS127A, silencing mouse *LAMC2* using shRNA (Figure S2G, Supporting Information) significantly reduced iCCA tumor formation (Figure 2F,G). In both models, all mice in control groups developed massive iCCA tumors (≥ 50 nodules), while mice in all sh*LAMC2* groups displayed reduced or even no iCCA tumor formation. Quantitatively, silencing *LAMC2* largely reduced the iCCA tumor burden, shown through remarkable decreases in liver/body ratios, tumor numbers, and tumor sizes in both iCCA models (*p* < 0.01 for each comparison). These results further support the oncogenic role of *LAMC2* in iCCA.

*LAMC2* is a subunit of the extracellular matrix protein laminin332 and a secretory protein. In iCCA cells, both endogenous and exogenous *LAMC2* could be secreted (Figure S3A,B, Supporting Information). Previous studies have reported *LAMC2* protein cleavage in its protein domain iii^[^ ^18^, ^19^ ^]^ (Figure S3C, Supporting Information). Therefore, we investigated the functional form of *LAMC2* protein in iCCA and found the full‐length intracellular *LAMC2* (rather than the secreted form) as the major functional form in regulating iCCA malignancy features (Figures S3 and S4, Supporting Information). First, endogenous *LAMC2* presented two main protein bands, a major band at ≈150 kDa (the full‐length) and a ≈105 kDa band (the long‐cleaved C‐terminal form) (Figure 1D). The major secreted *LAMC2* form was at ≈150 kDa (Figure S3A,B, Supporting Information). Second, *LAMC2* containing Flag‐tags at its domains v and iii consistently showed ≈150 kDa *LAMC2* as its major intracellular and secreted form (Figure S3D, Supporting Information). The presence of ≈105 kDa *LAMC2* in cell lysates was at least partially due to the mixed extracellular matrix on the cell membrane, as shown by a lower ≈105 kDa *LAMC2* level in cell lysates from the trypsin method (less extracellular matrix) than those from the scraping method (more extracellular matrix) (Figure S3E, Supporting Information). The data align with *LAMC2* cleavage occurring extracellularly.^[^ ^18^, ^19^ ^]^ Third, *LAMC2*‐high RBE cells showed increased cell proliferation and colony formation compared to *LAMC2*‐low RBE cells (Figure S4A,B, Supporting Information). However, the exposure of RBE cells to *LAMC2*‐high and *LAMC2*‐low conditioned medium did not lead to differences in cell proliferation and colony formation (Figure S4C, Supporting Information).

### 2.4 *LAMC2* Enhanced EGFR Protein Level by Promoting EGFR Translation

**Figure 3:**
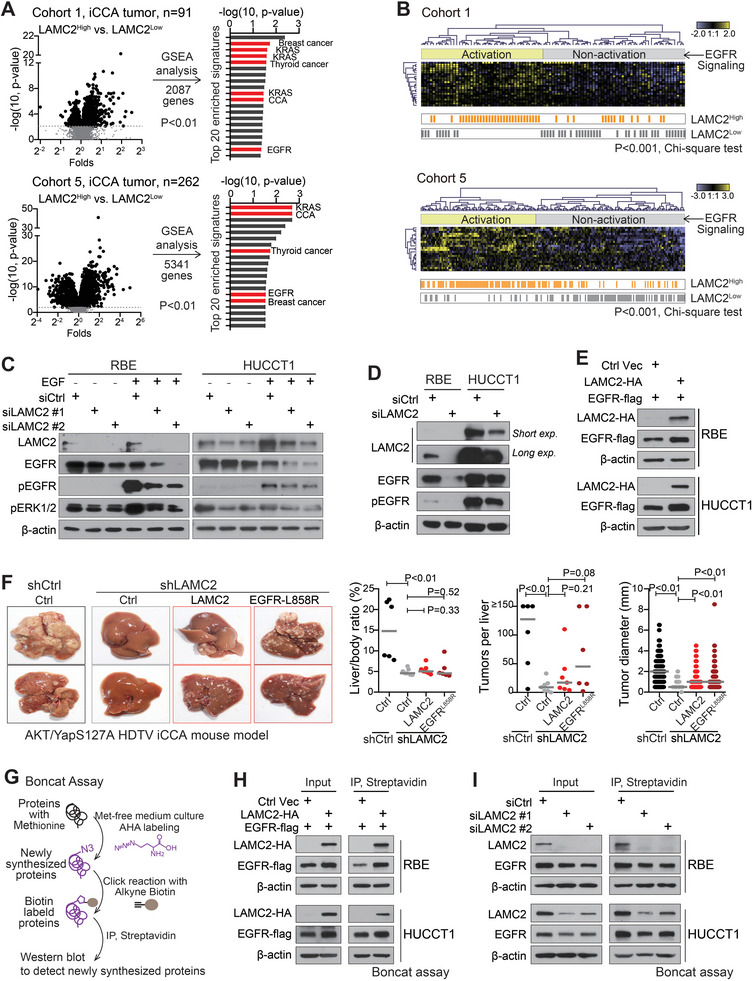
High *LAMC2* associated with EGFR signaling activation and *LAMC2* promoted EGFR translation. A) GSEA analysis with significantly altered genes (*p* < 0.01) between *LAMC2*‐high and *LAMC2*‐low iCCA patients. The top 20 enriched signatures were listed. B) The enrichment of *LAMC2*‐high patients in EGFR signaling activation and non‐activation groups subclassified by an EGF/EGFR signaling gene set. C) Western blot analysis in RBE and HUCCT1 cells transfected with control siRNA, si*LAMC2* #1, or si*LAMC2* #2 and treated with EGF. D) Western blot analysis in RBE and HUCCT1 cells transfected with control siRNA as well as si*LAMC2* #1 and #2. E) Western blot analysis of RBE and HUCCT1 cells transfected with Ctrl vector or *LAMC2*‐HA, along with EGFR‐flag. F) iCCA tumor formation in AKT/YapS127A‐induced iCCA mouse model with or without silencing *LAMC2* by shRNAs, upon with or without *LAMC2*/EGFR^L858R^ overexpression. Student's *t*‐test was used. G) The flow chart of Boncat Assay with L‐AHA to detect the newly synthesized proteins. H) Boncat assay in RBE and HUCCT1 cells with *LAMC2* overexpression. I) Boncat assay in RBE and HUCCT1 cells with *LAMC2* silencing.

To investigate the molecular mechanism of *LAMC2* in iCCA oncogenesis, Gene Set Enrichment Analysis (GSEA) was performed in iCCA tumors from Cohorts 1 and 5 (with >90 iCCAs in both cohorts) between the *LAMC2*‐high group and the *LAMC2*‐low group based on the median‐*LAMC2* cut‐off (**Figure** **3**A). Among the top 20 signatures identified from GSEA, several cancer‐related signatures, including the CCA signature and EGFR & KRAS signaling signatures, were significantly enriched in both cohorts (Figure 3A). Moreover, when iCCA patients were classified into EGFR signaling activation and inactivation groups based on EGF/EGFR signaling gene sets from literature,^[^ ^20^ ^]^ *LAMC2*‐high expression iCCA cases notably assembled in the EGFR signaling activation group of both cohorts (*p* < 0.001) (Figure 3B). Consistent results were obtained in mass spectrometry (MS) proteomic analysis with RBE cells with/without silencing *LAMC2* (Figure S5A,B, Supporting Information). GSEA analysis was performed with a significantly altered expression between the two groups. Among the top identified 20 signatures, two EGF/EGFR‐related signatures were enriched in the control group compared to the *LAMC2* silencing group (Figure S5C, Supporting Information). Meanwhile, protein intensities of genes presented in the EGF/EGFR signaling gene set including EGFR were also mainly reduced in the si*LAMC2* group (Figure S5D, Supporting Information).

Comparably, in both RBE and HUCCT1 cells, the silencing of *LAMC2* decreased the EGF‐mediated EGFR signaling activation as shown by reduced levels of phosphorylated EGFR and phosphorylated ERK (Figure 3C). More importantly, silencing *LAMC2* also reduced the EGFR protein level and the baseline of EGFR signaling activation considerably, shown by a reduced level of phosphorylated EGFR (Figure 3C,D). Comparably, *LAMC2* overexpression significantly increased the EGFR expression in both RBE and HUCCT1 cells (Figure 3E). In Cohort 5, EGFR and *LAMC2* protein levels were significantly positively correlated in 214 iCCA tumors with proteome data (*p* < 0.001, Figure S6A, Supporting Information). On the other hand, neither the over‐expression of EGFR nor the EGF treatment induced *LAMC2* expression (Figure S6B,C, Supporting Information). Secreted *LAMC2* had no effect on EGF‐mediated activation of EGFR signaling either (Figure S6D,E, Supporting Information).

In vivo, overexpressing either the human *LAMC2* or the active human EGFR^L858R^ both partially rescued sh*LAMC2*‐mediated tumor suppression in the AKT/YapS127A‐induced HDTV iCCA mouse model (Figure 3F). Collectively, these results suggested that intracellular *LAMC2* increased EGFR protein expression and EGFR was important in *LAMC2*‐mediated iCCA formation.

Mechanistically, *LAMC2* did not induce EGFR mRNA expression or increase its protein stability in iCCA cells (Figure S7A–E, Supporting Information). EGFR protein translation was thus examined using Boncat assay in combination with the click chemistry reaction, which could enable the detection of newly synthesized proteins (Figure 3G). This assay revealed that *LAMC2* overexpression increased the amount of newly synthesized EGFR (Figure 3H), while silencing *LAMC2* reduced it (Figure 3I). Thus, *LAMC2* increased EGFR protein expression by promoting its translation.

### 2.5 *LAMC2* Interacted with the Unglycosylated EGFR in Endoplasmic Reticulum

**Figure 4:**
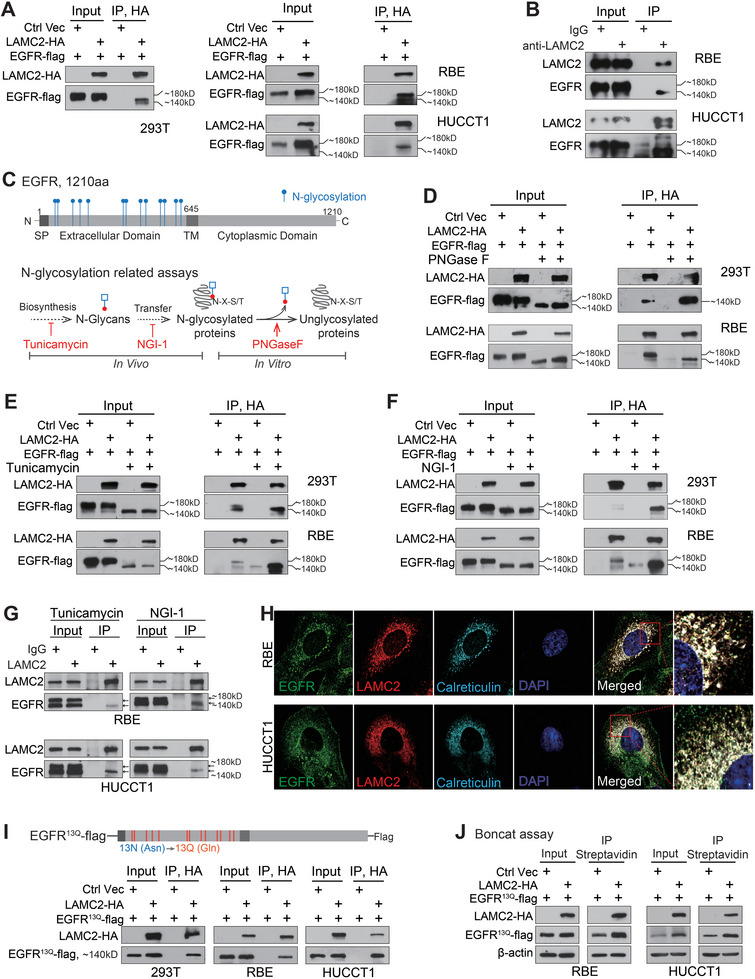
*LAMC2* interacted with unglycosylated EGFR. A) Cells were co‐transfected with indicated vectors and IP was performed with anti‐HA beads. B) IP assay with anti‐*LAMC2* antibody in iCCA cells. C) N‐linked glycosylation sites of EGFR (up panel) and the flow chart of the N‐glycosylation process (bottom panel). The related N‐glycosylation inhibitors and N‐glycan removing enzyme were indicated in red color. D) IP assay with anti‐HA beads in cells co‐transfected with *LAMC2*‐HA and EGFR‐flag. Both cell lysates and IP products were treated with or without PNGase F for 1 h before analysis. E) Anti‐HA IP in cells co‐transfected with *LAMC2*‐HA and EGFR‐flag and treated with or without tunicamycin (0.5 µg mL^−1^) for 24 h. F) Anti‐HA IP in cells co‐transfected with *LAMC2*‐HA and EGFR‐flag and treated with or without NGI‐1 (10 µm) for 24 h. G) Anti‐*LAMC2* IP in RBE and HUCCT1 cells treated with tunicamycin (0.5 µg mL^−1^) or NGI‐1 (10 µm) for 24 h. H) Confocal microscopy images of endogenous *LAMC2*, EGFR, and ER marker Calreticulin and their co‐localization in iCCA cells. I) Construction of EGFR^13Q^‐flag and anti‐HA IP assay in cells co‐transfected with *LAMC2*‐HA and EGFR^13Q^‐flag. J) Boncat assay in cells co‐transfected with *LAMC2*‐HA and EGFR^13Q^‐flag. SP, signal peptide; TM, transmembrane; N, Asparagine; Q, Glutamine.

In two iCCA cell lines and 293T, exogenously expressed *LAMC2*‐HA interacted with an undersized EGFR, which was ≈40 kDa smaller than the expected 180 kDa EGFR (**Figure** **4**A). Similarly, endogenous *LAMC2* also interacted with an undersized endogenous EGFR (≈140 kDa) in two iCCA cell lines (Figure 4B). Moreover, such an interaction was revealed in four out of five tested non‐iCCA cancer cell lines, and silencing *LAMC2* also reduced the EGFR protein translation, especially in pancreatic cancer cell line Panc‐1, lung cancer cell line H1975 and colorectal cancer cell line HCT‐116 (Figure S8A–C, Supporting Information).

EGFR is a transmembrane receptor tyrosine kinase that undergoes extensive asparagine (N)‐linked glycosylation in its extracellular domain, with 13 N‐glycosylation sites^[^ ^21^ ^]^ (Figure 4C). Each N‐linked glycosylation site contributes ≈3 kDa to the molecular weight of the modified protein. Therefore, the unglycosylated EGFR would be ≈40 kDa smaller compared to mature EGFR. We thus tested this possibility using several inhibitors targeting the different steps of the N‐linked glycosylation process, including peptide N‐glycosidase F (PNGase F) for removing N‐glycan chains from proteins, tunicamycin for blocking N‐glycan synthesizing, and NGI‐1 for inhibiting STT3 complex function in transferring N‐Glycans (Figure 4C).

As expected, upon the removal of N‐Glycans via PNGase F, the protein size of EGFR was ≈140 kDa. Meanwhile, a strong interaction was detected between exogenous *LAMC2* and the N‐glycan‐removed EGFR (≈140 kDa) (Figure 4D). Similar results were obtained when cells were treated with tunicamycin (Figure 4E) and NGI‐1 (Figure 4F). Treatment of tunicamycin and NGI‐1 led to the appearance of unglycosylated EGFR (≈140 kDa) and the *LAMC2*‐interacted EGFR remained at the size of ≈140 kDa. Meanwhile, blocking EGFR glycosylation with tunicamycin and NGI‐1 yielded a very strong interaction between *LAMC2* and EGFR (Figure 4E,F). Similar results were also obtained with the interaction of endogenous *LAMC2* and unglycosylated EGFR upon the treatment of tunicamycin and NGI‐1 (Figure 4G).

N‐linked glycosylation on proteins initiates in the endoplasmic reticulum (ER) lumen. Consistently, *LAMC2* and EGFR were clearly co‐localized with the ER marker calreticulin around the nucleus, shown by immuno‐fluorescence assay (Figure 4H). To further confirm the interaction of *LAMC2* with unglycosylated EGFR, an EGFR^13Q^‐flag construct was generated with N→Q mutations of all 13 glycosylation sites to mimic unglycosylated EGFR. The expressed EGFR^13Q^‐flag protein had a size of 140 kDa and strongly interacted with *LAMC2* (Figure 4I). Mature EGFR undergoes lysosomal‐related degradation upon ligand stimulation,^[^ ^22^ ^]^ while abnormally glycosylated proteins are commonly degraded via the ER‐associated protein degradation (ERAD) mechanism.^[^ ^23^ ^]^ Consistently, an ERAD inhibitor CB‐5083, but not a lysosome inhibitor Bafilomycin A1 rescued the degradation of EGFR^13Q^‐flag (Figure S9A,B, Supporting Information). As a control, Bafilomycin A1 but not CB‐5083 partially rescued mature EGFR degradation (Figure S9C, Supporting Information). Thus, the EGFR^13Q^‐flag fairly represented a nascent unglycosylated situation of EGFR and showed a strong interaction with *LAMC2*.

Comparably, neither knocking down *LAMC2* nor *LAMC2* overexpression affected the degradation of EGFR^13Q^‐flag (Figure S9D,E, Supporting Information). Nonetheless, *LAMC2* overexpression significantly increased the protein synthesis of EGFR^13Q^‐flag (Figure 4J). These data collectively suggested that *LAMC2* interacted with nascent or immature EGFR (≈140 kDa) and enhanced its translation.

### 2.6 *LAMC2* N‐Terminus Interacted with Extracellular Domain of EGFR, Promoting EGFR Translation

**Figure 5:**
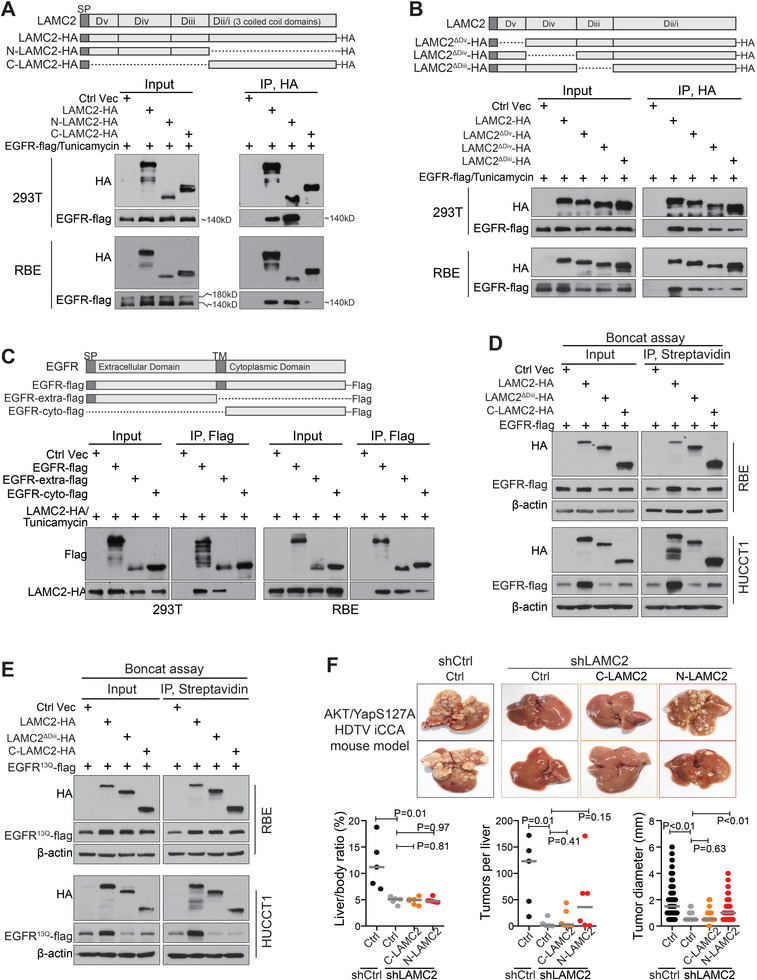
*LAMC2* N‐terminus interacted with the extracellular domain of EGFR, promoting EGFR translation. A) Mapping *LAMC2* regions involved in EGFR binding via IP in cells co‐transfected with EGFR‐flag and *LAMC2*‐HA deletion mutants. B) Mapping *LAMC2* N‐terminus regions involved in EGFR binding via IP in cells co‐transfected with EGFR‐flag and *LAMC2*‐HA deletion mutants. C) Mapping EGFR regions involved in *LAMC2* binding via IP in cells co‐transfected with *LAMC2*‐HA and different EGFR‐flag vectors. D) Boncat assay in cells co‐transfected with *LAMC2*‐HA deletion mutants and EGFR‐flag. E) Boncat assay in cells co‐transfected with *LAMC2*‐HA deletion mutants and EGFR^13Q^‐flag. F) iCCA tumor formation in AKT/YapS127A‐induced iCCA mouse model with or without silencing *LAMC2* by shRNAs, upon with or without C‐*LAMC2*/N‐*LAMC2* overexpression. Student's *t*‐test was used.

We further mapped the interaction regions of *LAMC2* and EGFR. Co‐IP assay revealed that the N‐terminus of *LAMC2* (N‐*LAMC2*, domains iii‐v), not the C‐terminus of *LAMC2* (C‐*LAMC2*, domains i‐ii), interacted with immature EGFR (**Figure** **5**A). Moreover, when domains iii‐v of N‐*LAMC2* were discretely removed (*LAMC2*‐ΔDv, *LAMC2*‐ΔDiv, *LAMC2*‐ΔDiii), each deletion resulted in a decreased interaction of *LAMC2* with immature EGFR (Figure 5B). The interaction reduction was particularly significant when domain iii or domain iv of *LAMC2* was removed. Thus, the N‐terminus of *LAMC2* was essential for interacting with EGFR. Next, Co‐IP assay showed that the extracellular region of EGFR interacted with *LAMC2*, while the cytoplasmic region of EGFR did not (Figure 5C). This interaction aligns with the current understanding that the EGFR extracellular region resides in the ER lumen.

Furthermore, although the intact *LAMC2* significantly increased the EGFR translation in both RBE and HUCCT1 iCCA cells, either *LAMC2* C‐terminus or *LAMC2*‐ΔDiii mutant (as an extra test) did not (Figure 5D). Thus, the interaction between *LAMC2* N‐terminus and EGFR was necessary for promoting EGFR translation. Consistent results were also obtained when EGFR^13Q^‐flag was co‐transfected with different *LAMC2* vectors (Figure 5E). Comparably in the AKT/YapS127A‐induced HDTV iCCA mouse model, overexpressing the N‐terminus of *LAMC2* partially rescued the suppressed iCCA carcinogenesis and progression caused by knocking down mouse *LAMC2*, but overexpressing *LAMC2* C‐terminus did not (Figure 5F). Taken together, the *LAMC2* N‐terminus interacted with the extracellular domain of EGFR during its immature status. This then promoted EGFR translation, contributing to iCCA development.

### 2.7 *LAMC2* Promoting EGFR Translation Was Partially Dependent on BiP, an ER Chaperon

**Figure 6:**
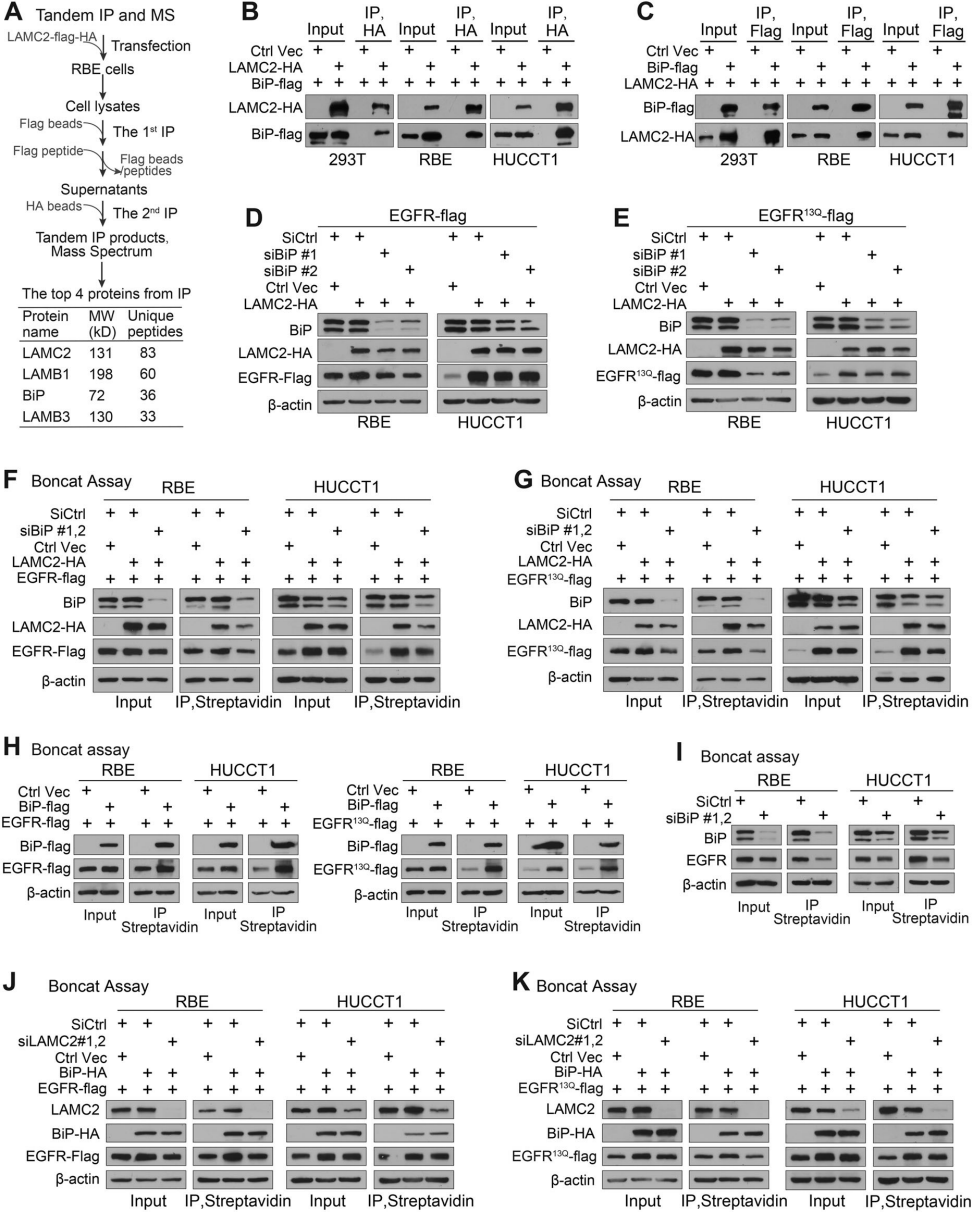
*LAMC2* promoting EGFR translation was partially dependent on BiP. A) The flow chart of tandem IP followed by MS, and the top 4 candidates were listed based on the quantity of unique peptides. B) Anti‐HA IP in cells co‐transfected with *LAMC2*‐HA and BiP‐flag. C) Anti‐flag IP in cells co‐transfected with *LAMC2*‐HA and BiP‐flag. D) EGFR‐flag expression in cells co‐transfected with *LAMC2*‐HA and EGFR‐flag with or without silencing BiP. E) EGFR^13Q^‐flag expression in cells co‐transfected with *LAMC2*‐HA and EGFR^13Q^‐flag with or without silencing BiP. F) Boncat assay in cells co‐transfected with *LAMC2*‐HA and EGFR‐flag with or without silencing BiP. G) Boncat assay in cells co‐transfected with *LAMC2*‐HA and EGFR^13Q^‐flag with or without silencing BiP. H) Boncat assay in cells transfected with BiP‐flag and EGFR‐flag (left panel) or EGFR^13Q^‐flag (right panel). I) Boncat assay in RBE and HUCCT1 cells upon BiP silencing. J,K) Boncat assay in cells co‐transfected with BiP‐HA and EGFR‐flag (J) or EGFR^13Q^‐flag (K) with or without silencing *LAMC2*.

A tandem IP followed by mass spectrometry (MS) was carried out to decode mechanisms of *LAMC2* in promoting EGFR translation. The top three identified *LAMC2* interacting proteins were LAMB1, BiP, and LAMB3 (**Figure** **6**A). LAMB1 and LAMB3 are the subunits of the laminin complex, with LAMB3 being a subunit of laminin332 along with *LAMC2*. BiP is a resident protein of the ER lumen. It is involved in assisting protein translation via binding to newly synthesized proteins as they are translocated into the ER lumen, and via counteracting the translation inhibitory effects induced by ER chaperon ERdj1, ERdj2/Sec62 and the ERdj6/PERK axis as BiP interacts with ERdj proteins with its nucleotide‐binding domain.^[^ ^24^ ^]^ We thus chose BiP for further investigation.

Co‐IP assays revealed a strong interaction between *LAMC2* with BiP upon their overexpression (Figure 6B,C). Moreover, silencing BiP in iCCA cell lines led to a reduction in EGFR protein expression promoted by *LAMC2* (Figure 6D). Comparable data were obtained when EGFR^13Q^ was used (Figure 6E). Boncat assay further showed that *LAMC2*‐mediated protein translation of EGFR and EGFR^13Q^ was visibly suppressed upon BiP silencing in both iCCA cells (Figure 6F,G). Thus, the *LAMC2*‐promoted EGFR translation was partially dependent on BiP.

Besides assisting in protein translation, BiP also recognizes unfolded/misfolded proteins in ER for ERAD degradation or for initiating the unfolded protein response, which reduces the level of targeted proteins.^[^ ^25^, ^26^ ^]^ However, overexpressing BiP did not reduce, but increased the protein levels of both EGFR and EGFR^13Q^ in iCCA cells (Figure 6H). In the same set of cells, BiP also increased the protein translation of both EGFR and EGFR^13Q^ (Figure 6H). Consistent data were obtained when endogenous BiP was silenced (Figure 6I). In this case, BiP mainly promoted EGFR translation.

In addition, when *LAMC2* was silenced, BiP‐promoted EGFR translation was observably weakened (Figure 6J,K), indicating that the promotion of EGFR translation by BiP was also dependent on *LAMC2*. Collectively, *LAMC2* and BiP increased EGFR protein translation interdependently.

### 2.8 *LAMC2*, EGFR, and BiP Interacted with Each Other, Contributing to EGFR Translation

**Figure 7:**
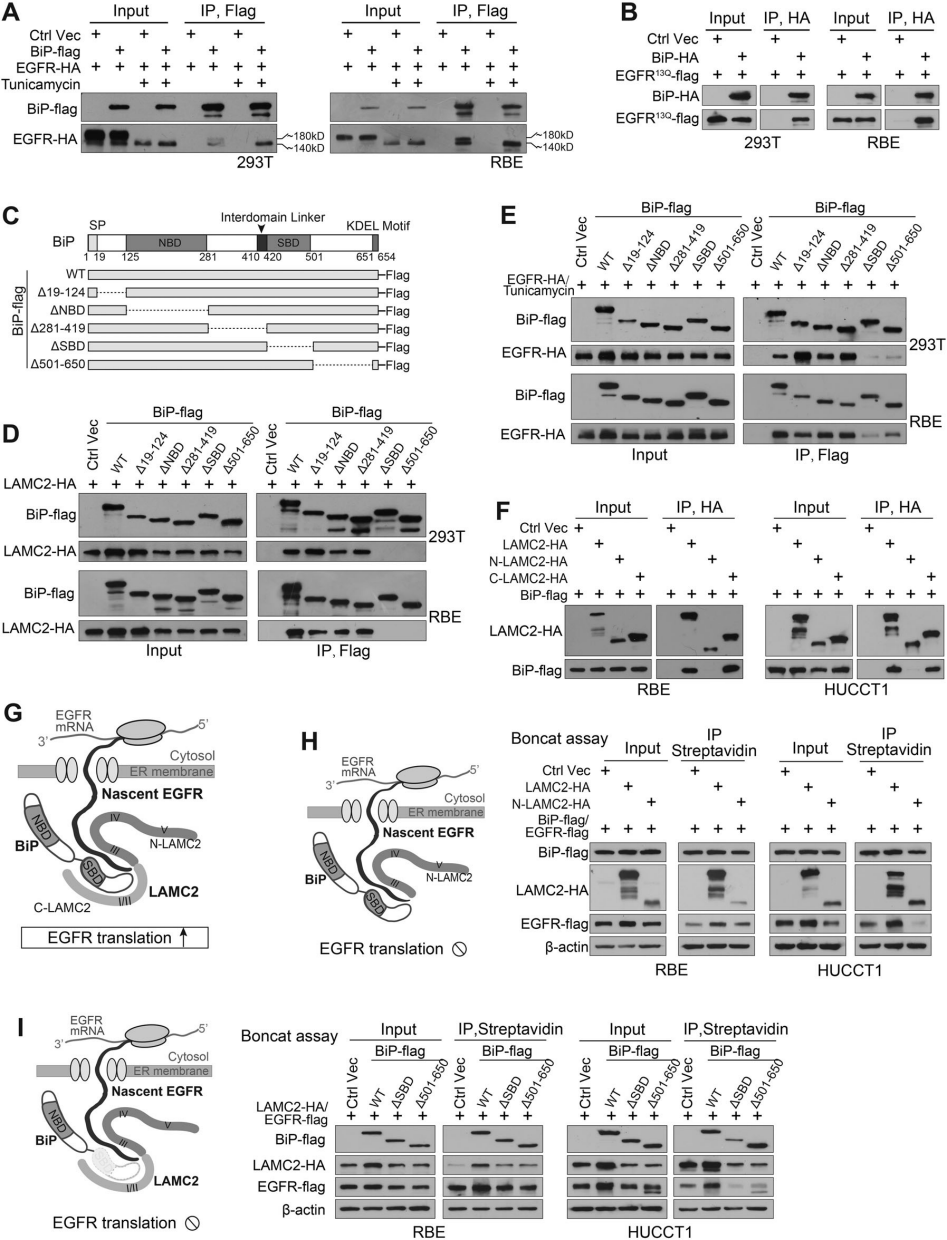
*LAMC2*, EGFR, and BiP interacted with each other, contributing to EGFR translation. A) Anti‐flag IP in cells co‐transfected with BiP‐flag and EGFR‐HA with or without tunicamycin treatment. B) Anti‐HA IP in cells co‐transfected with BiP‐HA and EGFR^13Q^‐flag. C) Schematic diagram of BiP functional domain and a group of BiP truncations. D) Mapping BiP regions involved in *LAMC2* binding via IP in cells co‐transfected with *LAMC2*‐HA and different BiP‐flag vectors. E) Mapping BiP regions involved in EGFR binding via IPs in cells co‐transfected EGFR‐HA and different BiP‐flag vectors with tunicamycin treatment. F) Anti‐HA IP in cells co‐transfected with BiP‐flag and different *LAMC2* vectors. G) An illustrated interacting model of *LAMC2*, BiP, and nascent EGFR in promoting EGFR translation in ER. H) Boncat assay in cells co‐transfected with BiP‐flag/EGFR‐flag and an intact *LAMC2*, or N‐*LAMC2*. I) Boncat assay in cells co‐transfected with *LAMC2*‐HA /EGFR‐flag and an intact BiP, or BiP^ΔSBD^ and BiP^Δ501‐650^. SP, signal peptide; NBD, nucleotide‐binding domain; SBD, substrate binding domain.

The Co‐IP assay revealed two EGFR bands interacting with BiP, a full‐size EGFR and an undersized ≈140 kDa EGFR, which was similar to EGFRs interacting with *LAMC2* (**Figure** **7**A). Concurrently, ≈140 kDa EGFR remained as the major band to interact with BiP when cells were exposed to tunicamycin (Figure 7A), and BiP also strongly interacted with EGFR^13Q^ (Figure 7B). In this case, not only *LAMC2* but also BiP interacted with nascent EGFR without N‐glycosylation.

BiP contains a signal peptide (1–18aa), a nucleotide‐binding domain (NBD, 125–280aa), a substrate‐binding domain (SBD, 420–500aa), and an ER retention KDEL motif. Co‐IP assay with BiP truncations revealed that the removal of either SBD or 501–650aa region of BiP completely abrogated the interaction of BiP with *LAMC2* (Figure 7D). These two regions were also crucial for BiP to interact with EGFR (Figure 7E). Moreover, the C‐terminus of *LAMC2*, yet not the N‐terminus, interacted with BiP (Figure 7F). In line with these findings, the extracellular domain of EGFR interacted with BiP (Figure S10, Supporting Information). These suggested the importance of BiP C‐terminus in EGFR translation. Based on these data, a possible interacting model of *LAMC2*, BiP, and EGFR was proposed (Figure 7G). *LAMC2* C‐terminus interacts with the BiP C‐terminal region (including its SBD domain and region 501–650aa) in the ER, forming a “pocket” composed of the BiP C‐terminus and *LAMC2* N‐terminus. This “pocket” binds to nascent EGFR at its extracellular domain, leading to an increased EGFR translation.

This model was further validated via several Boncat assays after this “pocket” was disrupted. As expected, overexpression of wild‐type *LAMC2* increased the levels of newly synthesized EGFR or EGFR^13Q^ in the presence of high levels of BiP expression, whereas *LAMC2* lacking C‐terminus did not have this effect (Figure 7H; Figure S11A, Supporting Information). Moreover, wild‐type BiP enhanced the levels of newly synthesized EGFR or EGFR^13Q^, but BiP mutants lacking the region 501–650aa or the SBD domain could not (Figure 7I; Figure S11B, Supporting Information).

### 2.9 *LAMC2*‐High iCCA Tumors Had Poor Prognosis but Were Sensitive to EGFR TKIs Treatment

**Figure 8:**
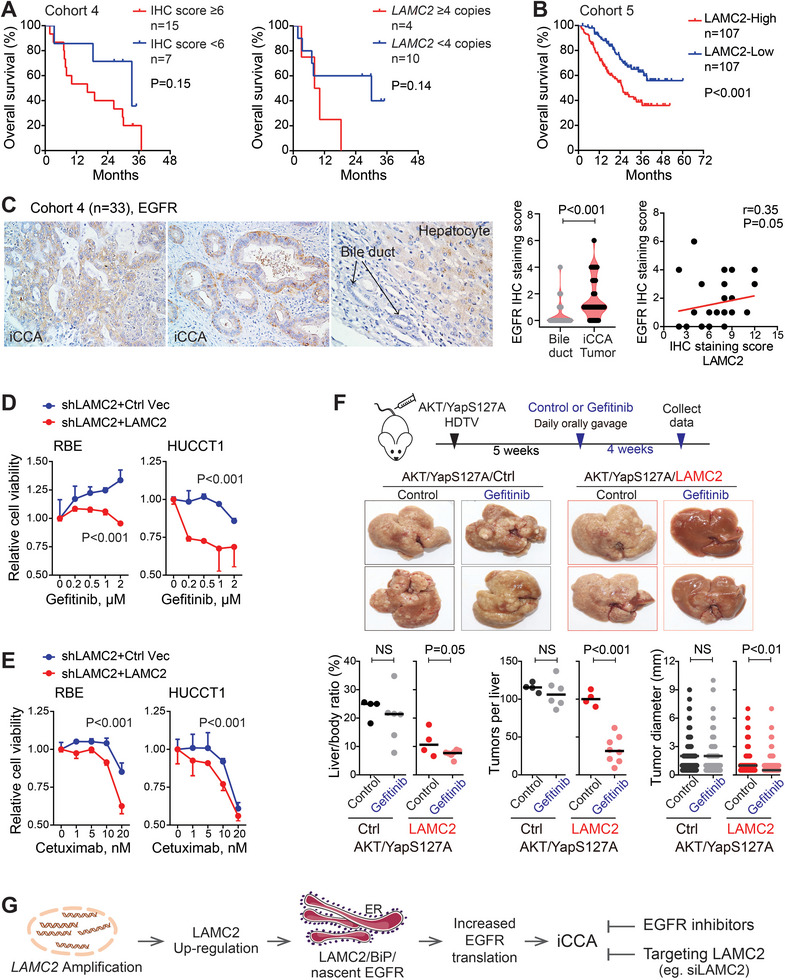
*LAMC2*‐high iCCA tumors had poor prognosis, but were sensitive to EGFR TKIs treatment. A) Kaplan–Meier survival analysis of iCCA patients from Cohort 4 based on *LAMC2* IHC staining score and *LAMC2* copy number. B) Kaplan–Meier survival analysis of iCCA patients from Cohort 5 based on *LAMC2* protein level (the median cut‐off). C) EGFR IHC staining and the spearman correlation of EGFR and *LAMC2* in Cohort 4. Student's *t*‐test was used for group comparison. D,E) Cell viability of iCCA cells with different *LAMC2* levels under treatment of EGFR TKI Gefitinib (C) or EGFR neutralizing antibody Cetuximab (D). Two‐way ANOVA analysis was used. F) Representative images and quantitative analysis of orthoptic iCCA tumor formation in AKT/YapS127A‐induced iCCA mouse model with or without *LAMC2*, upon with or without Gefitinib treatment. Student's *t*‐test was used. NS, not significant. G) The schematic model summarized *LAMC2* as a key oncogenic event in iCCA.

With the limited number of iCCA patients in Cohort 4, *LAMC2*‐high cases (IHC score ≥6) and *LAMC2*‐amplification cases (copy number ≥4) appeared to have shorter overall survival with borderline statistical P‐values compared to their corresponding control groups (**Figure** **8**A). Whereas, neither *LAMC2* staining nor *LAMC2* amplification in this cohort was significantly related to other clinical parameters (Table S3, Supporting Information). Comparable and much more significant data were obtained in Cohort 5 with over 200 iCCA patients. In this cohort, patients with high‐*LAMC2* protein levels in their iCCA tumors showed significantly shorter overall survival compared to iCCA cases having low‐*LAMC2* levels, based on various cut‐offs of *LAMC2* protein level (median, tertile, or quartile division) (Figure 8B; Figure S12A–C, Supporting Information, *p* < 0.001 for each comparison).

Consistent with *LAMC2* promoting EGFR translation, EGFR IHC staining in Cohort 4 showed a significantly higher staining score in iCCA tumors compared to bile ducts (*p* < 0.001) and a positive correlation with *LAMC2* staining in iCCA tumor tissues (*p* = 0.05) (Figure 8C). We then tested the sensitivity of *LAMC2*‐high iCCA cells to EGFR TKIs treatment. In response to EGFR TKI Gefitinib and EGFR neutralizing antibody Cetuximab, cell viability was lower in *LAMC2*‐high cells (sh*LAMC2*+*LAMC2*) in comparison to *LAMC2*‐low cells (sh*LAMC2*+Ctrl Vec) of two iCCA cell lines (Figure 8D,E), indicating that *LAMC2*‐high iCCA cells were more sensitive to EGFR TKIs. This result was further evaluated in vivo using AKT/YapS127A‐induced HDTV iCCA mouse model (Figure 8F). Two sets of mice were prepared, i.e., one injected with AKT/YapS127A, and the other injected with AKT/YapS127A and *LAMC2*. Five weeks after injection, each set was assigned randomly to the treatment group accepting Gefitinib and the control group accepting the sterilized ultrapure water. The results showed that mice in the AKT/YapS127A/*LAMC2* control group developed observable massive tumors, while AKT/YapS127A/*LAMC2* mice with Gefitinib treatment had much less tumor formation. Significant quantitative data were also obtained on the liver/body ratios and tumor numbers (Figure 8F). This difference was not noticed in AKT/YapS127A‐induced HDTV iCCA tumors. Taken together, *LAMC2*‐high iCCA tumors with poor prognosis were sensitive to EGFR TKIs treatment.

## 3 Discussion

In this study, our results revealed *LAMC2* as a new oncogenic player in iCCA patients and illustrated a signaling axis of *LAMC2*→ *LAMC2*/BiP/nascent EGFR→EGFR translation→iCCA carcinogenesis (Figure 8G). In this axis, a high level of *LAMC2*, partially caused by *LAMC2* amplification in iCCA, increased EGFR translation, which in turn promoted iCCA tumorigenesis and demonstrated the sensitivity of *LAMC2*‐high iCCAs to EGFR TKIs treatment.

As a laminin family member, *LAMC2* was known to regulate cell invasion, migration, and tumor metastasis in several cancers including CCA.^[^ ^14^, ^17^, ^27^ ^]^ Meanwhile, several recent studies reported its role in promoting cell proliferation in ovarian cancer and pancreatic cancer.^[^ ^28^, ^29^ ^]^ Mechanistically, few of these studies suggested a link between *LAMC2* and AKT signaling and a possible regulation between EGFR signaling and *LAMC2*.^[^ ^14^, ^30^, ^31^, ^32^ ^]^ However, the mechanism remained unknown on whether and how EGFR signaling and *LAMC2* regulated each other. Here we found that silencing *LAMC2* reduced cell migration but more significant results were observed on its blocking iCCA formation in vivo. Mechanistically, we revealed thoroughly that *LAMC2* significantly promoted EGFR translation. *LAMC2* and BiP interacted via their C‐terminus, creating a “pocket” composed of *LAMC2* N‐terminus and BiP C‐terminus. This pocket captured newly synthesized EGFR without glycosylation and promoted EGFR translation. Meanwhile, it was not only in iCCA cells but also in several non‐iCCA cancer cell lines that *LAMC2* promoted EGFR translation via interacting with ≈140 kD EGFR. Thus, our findings attributed *LAMC2* to a new function in promoting EGFR translation across different cancer cell lines. Moreover, it is likely that *LAMC2* might promote not only EGFR translation but also other proteins. Further in‐depth investigations are ongoing to uncover more breakthrough findings in this regard.

BiP was previously known to support protein translation via its NBD domain, by which it interacted with ERdj proteins and abolished the translation inhibition mediated by ERdj1, ERdj2/Sec62, or the ERdj6/PERK axis.^[^ ^24^ ^]^ Consistently, these known mechanisms of BiP contributed to EGFR translation too (Figure S13, Supporting Information), i.e., BiP's NBD domain was important for BiP promoting EGFR translation; overexpression of ERdj1 or ERdj2 consistently reduced BiP‐mediated EGFR protein translation; silencing PERK rescued the decrease in EGFR protein translation caused by BiP silencing. We have revealed here the importance of the BiP C‐terminal region in EGFR translation (Figure 7), which thus extended the mechanism of BiP in protein translation. It will be interesting to further investigate whether the BiP C‐terminal region functions in other proteins’ translation and whether such a mechanism mainly relies on *LAMC2*. In iCCA, *LAMC2* was significantly up‐regulated whereas BiP expression did not seem to be deregulated across different CCA cohorts (Figure S14A, Supporting Information). BiP levels in iCCA tumors were not related to iCCA prognosis either or EGFR signaling activation (Figure S14B,C, Supporting Information). Therefore, *LAMC2* appeared to be the key leading factor and BiP was jointly involved in promoting EGFR protein synthesis, at least in iCCA.

In clinics, several clinical trials have been performed to investigate the use of EGFR TKIs in treating CCA patients, but the results have been disappointing. In this study, a tight relationship was established between the level of *LAMC2* and EGFR signaling activation. In this case, *LAMC2* might be a valuable indicator to guide the EGFR TKI treatment in clinical practice for iCCA patients. More interestingly, *LAMC2* could be secreted from iCCA cells. Therefore, it is worthwhile to further determine and consider the serum *LAMC2* level as a non‐invasive biomarker to stratify iCCA patients with high *LAMC2* levels either for EGFR TKIs therapy alone or concurrently with systemic chemotherapy.

Moreover, the current strategies for targeting EGFR mainly focus on targeting the kinase domain of EGFR or binding the extracellular domain of EGFR to prevent ligand binding or receptor dimerization. Our finding of *LAMC2* interacting with an immature EGFR without glycosylation highlighted the presence of nascent EGFR before its undergoing glycosylation and maturation in ER. It therefore offers a new opportunity to develop methods that target nascent EGFR or block its translation, consequently reducing the amount of mature EGFR and the EGFR signaling activation.

Exploring the potential of targeting *LAMC2* as a therapeutic approach in iCCA is an intriguing avenue for further research. However, it is important to approach this with caution. Mutations in all three laminin332 subunit chains caused skin disease junctional epidermolysis bullosa (JEB). Knockout mice lacking any of the three chains exhibited symptoms similar to human JEB and died within a few days after birth.^[^ ^33^, ^34^ ^]^ Considering these phenotypes observed in *LAMC2*‐knockout animals, it might be feasible to further investigate knocking down *LAMC2*, as we have done in this manuscript, or suppressing *LAMC2*’s function in ER via other methods as the safe and suitable strategies to treat iCCA.

## 4 Conclusion

In summary, this study identified *LAMC2* as a key oncogenic molecule in iCCA and highlighted its potential as an indicator for guiding EGFR TKI treatment in clinical practice. Amplification of *LAMC2* gene in iCCA led to increased levels of *LAMC2* protein. Within the ER, the increased *LAMC2* together with BiP interacted with newly synthesized EGFR, promoting EGFR translation. Consequently, the *LAMC2*/EGFR axis contributed to iCCA carcinogenesis, and iCCA tumors with high levels of *LAMC2* expression exhibited sensitivity to EGFR TKIs treatments.

## 5 Experimental Section

### iCCA Cohorts, Omics Datasets, and Other Data Sources

A total of five cohorts were used in this study (Table S1, Supporting Information). Cohort 1 included 91 iCCA patients and 62 HCC patients with available paired tumor and non‐tumor mRNA array transcriptome data (GSE76297). Cases in this cohort were from Thailand in Asia. Cohort 2 included 36 CCA cases (86% of iCCAs) with RNA sequencing data in all CCA tissues and 9 non‐tumor tissues from the Cancer Genome Atlas (TCGA). Cases in cohort 2 were mainly Caucasian. Cohort 3 consisted of 104 CCA cases (65% of iCCAs), 59 surrounding liver tissues, and 6 normal bile duct tissues with available mRNA array transcriptome data (GSE26566). Cases in this cohort were mainly Caucasians from the United States, Belgium, and Australia. Cohort 4 consisted of 33 iCCA patients and 20 HCC patients with available archived formalin‐fixed paraffin‐embedded (FFPE) iCCA and HCC tissues. They were from Shandong Cancer Hospital and Institute in China, and the institutional review board approved the use of these FFPE tissues and waived the requirement for informed consent. The related information was also summarized in Table S2 (Supporting Information). Cohort 5 included 262 iCCA patients with tumor and adjacent non‐tumor tissues, among which 255 patients had available tumor RNA transcriptome data and 214 patients had available tumor proteome data.^[^ ^35^ ^]^ These iCCA patients were all Chinese.

In addition, available *LAMC2* amplification frequency was collected from cBioPortal database (http://www.cbioportal.org/), which included 10 953 patients (10 967 samples) from 33 cancer types in a total of 32 studies (TCGA PanCancer Atlas Studies). The EGF/EGFR signaling gene sets including 23 genes were from Dr. Hung's study in Cancer Cells and were used in hierarchical clustering analysis.^[^ ^20^ ^]^ Available *LAMC2* IHC staining results in tumor tissues were collected from the Human Protein Atlas database (https://www.proteinatlas.org/).

### Cell Culture and Treatment

Human iCCA cell lines (RBE, HUCCT1, Huh28) and human lung cancer cell lines (A549, H1975) were maintained in RPMI 1640 medium supplemented with 10% fetal bovine serum, 100 U mL^−1^ penicillin–streptomycin and 1% L‐glutamine. Human HCC cell lines (Huh1, Huh7, HLE, HLF), human pancreatic cancer cell line Panc‐1, human colorectal cancer cell line HCT‐116, human breast cancer cell line MDA‐MB‐231, human embryonic kidney cell line 293T and mouse primary hepatocyte cell line H2.35 were maintained in Dulbecco's modified Eagle's medium (DMEM) supplemented with 10% fetal bovine serum, 100 U mL^−1^ penicillin–streptomycin and 1% L‐glutamine. RBE and Panc‐1 cells were from the Cell Bank of the Chinese Academy of Sciences (Shanghai, China). HUCCT1, Huh28, and four HCC cell lines were from Japanese Collection of Research Biosources Cell Bank (JCRB). HCT‐116, 293T, and H2.35 cell lines were originally from American Type Culture Collection (ATCC). A549 and H1975 cells were kindly provided by Dr. Hai Song and MDA‐MB‐231 cells were by Dr. Weijie Zhang in our institute. Peripheral blood mononuclear cells (PBMCs) were from three healthy donors and used as controls to examine the *LAMC2* gene copy number.

Upon the EGF treatment, cells were treated with 100 ng mL^−1^ EGF (Cat# AF‐100‐15, PeproTech) for 10–30 min after overnight starvation as indicated in the manuscript. To detect protein stability, iCCA cells were treated with 20 µg mL^−1^ cycloheximide (CHX, Cat# 2112S, Cell signaling technology) and lysed at the indicated time after CHX addition. Cells were also treated with 20 µg mL^−1^ CHX and 100 nm bafilomycin A1 (Cat# HY‐100558, MedChemExpress) for 12 h to measure EGFR degradation, as well as with 20 µg mL^−1^ CHX and 2 µm CB‐5083 (Cat# HY‐12861, MedChemExpress) to examine the degradation of unglycosylated EGFR. 20 µM MG132 (Cat# S2619, Selleck) and 2 µm CB‐5083 were used to confirm the proteosome‐related degradation and ER‐associated degradation, respectively. Moreover, 0.5 µg mL^−1^ tunicamycin (Cat# S7894, Selleck) and 10 µm NGI‐1 (Cat# S8750, Selleck) were used to treat cells for 12–24 h to block protein glycosylation.

### DNA Extraction and Copy Number Assay

Total genomic DNAs from FFPE tissues were extracted with MasterPure Complete DNA and RNA Purification Kit (Cat# MC85200, Epicentre) according to the manufacturer's instructions. Genomic DNAs from iCCA cell lines and PBMCs were extracted with the conventional phenol‐chloroform DNA extraction method. *LAMC2* copy number was detected with TaqMan Copy Number Assay (Cat# 4 400 291, Hs06577731_cn, Applied Biosystems) with TaqMan Genotyping Master Mix (Cat# 4 371 353, Applied Biosystems) according to the manufacturer's instructions. Human *RNase P* was used as a reference gene, known to exist in two copies in a diploid genome, and detected with TaqMan Copy Number Reference Assay (Cat# 4 403 326, Applied Biosystems). *LAMC2* copy numbers in test samples were determined by relative quantitation using the comparative Ct (ΔΔCt) method, which measured the C_t_ difference (ΔC_t_) between *LAMC2* and reference gene *RNase P*.

### Plasmids and siRNAs

Vectors pT3‐EF1α‐myr‐AKT, pT3‐EF1α‐YapS127A, pT3‐EF1α‐NICD, pT3‐EF1α‐Myc, NRasV12/pT2‐CAGGS and pCMV/Sleeping Beauty transposase (pCMV/SB) were constructed and used as previously described^[^ ^36^, ^37^, ^38^ ^]^ pT3‐EF1α‐*LAMC2* vector was generated via recombining *LAMC2* entry clone with a destination vector pT3‐EF1α‐attR‐ccdb using the Gateway LR Clonase II Enzyme mix (Cat# 11 791 020, Thermo Fisher Scientific). Vectors pT3‐EF1α‐C‐*LAMC2*, pT3‐EF1α‐N‐*LAMC2*, and pT3‐EF1α‐EGFR^L858R^ were constructed by inserting C‐*LAMC2*, N‐*LAMC2*, and EGFR^L858R^ into pT3‐EF1α‐attR‐ccdb vector using ClonExpress MultiS One Step Cloning Kit. pcDNA3.0‐*LAMC2*‐HA, pcDNA3.0‐ERdj1‐HA and pcDNA3.0‐ERdj2‐HA were constructed via inserting the *LAMC2* cDNA, ERdj1 cDNA and ERdj2 cDNA into BamHI/XhoI sites of pcDNA3.0‐HA vector, respectively. For the construction of pcDNA3.0‐N‐*LAMC2*‐HA, pcDNA3.0‐C‐*LAMC2*‐HA, *LAMC2* deletion mutants (pcDNA3.0‐*LAMC2* ^ΔDv^‐HA, pcDNA3.0‐*LAMC2* ^ΔDiv^‐HA, pcDNA3.0‐*LAMC2* ^ΔDiii^‐HA), pcDNA3.0‐EGFR‐HA, and pcDNA3.0‐BiP‐HA, the related *LAMC2*, EGFR and BiP cDNA fragments were inserted into pcDNA3.0‐HA vector using the ClonExpress MultiS One Step Cloning Kit (Cat# C113‐02, Vazyme). For flag‐tagged constructs p3xflag‐cmv‐14‐EGFR, p3xflag‐cmv‐14‐EGFR‐extra, and p3xflag‐cmv‐14‐EGFR‐cyto, the related EGFR cDNAs were subcloned into HindIII/XbaI sites of p3xflag‐cmv‐14 vector. For the construction of p3xflag‐cmv‐14‐EGFR^13Q^, EGFR with 13 glycosylation sites N mutated to Q was synthesized and inserted into the p3xflag‐cmv‐14 vector using ClonExpress MultiS One Step Cloning Kit. For flag‐tagged pcDNA3.0‐BiP‐flag and BiP deletion mutants (pcDNA3.0‐BiP^Δ19‐124^‐flag, pcDNA3.0‐BiP^ΔNBD^‐flag, pcDNA3.0‐BiP^Δ281‐419^‐flag, pcDNA3.0‐BiP^ΔSBD^‐flag, and pcDNA3.0‐BiP^Δ501‐650^‐flag), BiP cDNAs and flag tag were inserted into pcDNA3.0 vector with ClonExpress MultiS One Step Cloning Kit. To construct pcDNA3.0‐*LAMC2*‐flag‐HA, pcDNA3.0‐*LAMC2*‐V‐flag‐HA, and pcDNA3.0‐*LAMC2*‐III‐flag‐HA, flag tag was inserted into pcDNA3.0‐*LAMC2*‐HA vector by a homologous recombination reaction. To construct pLKO.1‐shCtrl, pLKO.1‐sh*LAMC2*#1, pLKO.1‐sh*LAMC2*#2, pLKO.1‐sh*LAMC2*#m1 and pLKO.1‐sh*LAMC2*#m2, the corresponding DNA fragments were synthesized including shCtrl, sh*LAMC2*#1, sh*LAMC2*#2, sh*LAMC2*#m1, and sh*LAMC2*#m2, and then were inserted into EcoRI/AgeI sites of pLKO.1 vector. Vectors pT3‐EF1α‐myr‐AKT‐shCtrl, pT3‐EF1α‐myr‐AKT‐sh*LAMC2*#m1 and pT3‐EF1α‐myr‐AKT‐sh*LAMC2*#m2 were constructed by inserting shCtrl, sh‐m*LAMC2*#1 and sh‐m*LAMC2* #2 into pT3‐EF1α‐myr‐AKT vector using ClonExpress MultiS One Step Cloning Kit. Lentiviruses were packaged with plasmids psPAX2 and pMD2.G (Addgene) in 293 T cells. For infection, 5 MOI of each lentivirus was used for all the studies.

*LAMC2* siRNAs, *BiP* siRNAs, *PERK* siRNAs, and scramble negative control siRNAs were purchased from GenePharma Co., Shanghai, China. Lipofectamine 2000 Reagent (Cat# 11 668 019, Invitrogen, US) and Rfect siRNA Transfection Reagent (Cat# 11 011, BIOTRAN) were used for transfections of plasmids and siRNAs, respectively. The detailed information for all primers used for constructs and siRNA targeting sequences is listed in Table S5 (Supporting Information).

### RNA Extraction and Quantitative Real‐Time PCR

Total RNA was extracted using TRIzol RNA isolation Reagents (Invitrogen) following the manufacturer's instructions. One microgram of total RNA was reverse transcribed to cDNA using PrimeScript RT reagent Kit (Cat# RR047, TaKaRa). Quantitative reverse transcription polymerase chain reaction (qRT‐PCR) was performed with the TB Green Premix Ex Taq II (Cat#RR420, TaKaRa). 18S was used as a reference gene. All primer sequences are listed in Table S5 (Supporting Information).

### Mouse Studies

All mouse procedures were conducted under the guidelines and the institutional animal care protocol approved by the Experimental Animal Committee at Zhejiang University. ICR mice were purchased from Shanghai SLAC Laboratory Animal Co.Ltd. FVB/N mice were from Beijing Vital River Laboratory Animal Technology. All mice were housed in Zhejiang University Laboratory Animal Center in laminar‐flow cabinets under specific pathogen‐free conditions at room temperature with a 24‐h night‐day cycle. Oncogene‐induced orthotopic iCCA and HCC mouse models were used. For these models, we performed hydrodynamic tail vein injection as we did before in six‐week‐old ICR or FVB/N mice^[^ ^39^ ^]^ with the related oncogenes and the sleeping beauty (SB) transposon system. Briefly, the combination of pT3‐EF1α‐myr‐AKT and pT3‐EF1α‐YapS127A (AKT/YapS127A), or the combination of pT3‐EF1α‐myr‐AKT and pT3‐EF1α‐NICD (AKT/NICD) along with pCMV/SB was introduced to induce iCCA formation through hydrodynamic tail vein injection. pT3‐EF1α‐Myc or the combination pT3‐EF1α‐myr‐AKT/NRasV12/pT2‐CAGGS (AKT/Ras) along with pCMV/SB was introduced to induce HCC formation.

To detect *LAMC2* mRNA expression, tumor, and non‐tumor liver tissues were collected from AKT/YapS127A‐induced iCCA mouse model (male, *n* = 5, sacrificed 6 weeks post‐injection; female, *n* = 6, sacrificed 8 weeks post‐injection), AKT/NICD‐induced iCCA mouse model (male, *n* = 4; female, *n* = 2; sacrificed 4 weeks post‐injection), AKT/Ras‐induced HCC mouse model group (female, *n* = 3; sacrificed 6 weeks post‐injection), c‐myc‐induced HCC mouse model (female, *n* = 3; sacrificed 8.5 weeks post‐injection), as well as mouse liver without oncogene injection (female, *n* = 4, sacrificed at 12‐week‐old). FVB/N mice were used.

To test silencing *LAMC2* in regulating iCCA formation, pT3‐EF1α‐myr‐AKT‐shCtrl, pT3‐EF1α‐myr‐AKT‐sh*LAMC2*#m1, and pT3‐EF1α‐myr‐AKT‐sh*LAMC2*#m2 were used in both AKT/NICD‐induced iCCA mouse model and AKT/YapS127A‐induced iCCA mouse model. In this assay, ICR female mice were used with 5–7 mice/group, i.e., AKT/NICD/shCtrl, *n* = 7; AKT/NICD/sh*LAMC2*#m1, *n* = 6; AKT/NICD/sh*LAMC2*#m2, *n* = 7; AKT/YapS127A/shCtrl, *n* = 6; AKT/YapS127A/sh*LAMC2*#m1, *n* = 5; and AKT/YapS127A /sh*LAMC2*#m2, *n* = 7. ICR mice were used. AKT/NICD‐induced mice were sacrificed 4.5 weeks post‐injection, and AKT/YapS127A‐induced mice were sacrificed 11.5 weeks post‐injection.

For *LAMC2* and EGFR rescue assay, pT3‐EF1α‐myr‐AKT‐shCtrl, pT3‐EF1α‐myr‐AKT‐sh*LAMC2*#m1, pT3‐EF1α‐*LAMC2*, and pT3‐EF1α‐EGFR^L858R^ were used in AKT/YapS127A‐induced iCCA mouse model. Four groups of female ICR mice were used, i.e., AKT‐shCtrl/YapS127A/Ctrl, *n* = 6; AKT‐sh*LAMC2*#m1/YapS127A/Ctrl, *n* = 7; AKT‐sh*LAMC2*#m1/YapS127A/*LAMC2*, *n* = 7 and AKT‐sh*LAMC2*#m1/YapS127A/EGFR^L858R^, *n* = 6. These mice were sacrificed 11 weeks post‐injection.

For C‐*LAMC2* and N‐*LAMC2* rescue assay, pT3‐EF1α‐myr‐AKT‐shCtrl, pT3‐EF1α‐myr‐AKT‐sh*LAMC2*#m1, pT3‐EF1α‐C‐*LAMC2*, and pT3‐EF1α‐N‐*LAMC2* were used in AKT/YapS127A‐induced iCCA mouse model. Four groups of female ICR mice were used, i.e., AKT‐shCtrl/YapS127A/Ctrl, *n* = 5; AKT‐sh*LAMC2*#m1/YapS127A/Ctrl, *n* = 5; AKT‐sh*LAMC2*#m1/YapS127A/C‐*LAMC2*, *n* = 6; and AKT‐sh*LAMC2*#m1/YapS127A/N‐*LAMC2*, *n* = 6. These mice were sacrificed 11 weeks post‐injection.

For Gefitinib treatment assay, ICR mice and AKT/YapS127A‐induced iCCA mouse model were used. Gefitinib was solved in sterilized ultrapure water. Five weeks after the oncogene injection, 150 mg kg^−1^ Gefitinib or an equal volume of sterilized ultrapure water was administrated by oral gavage daily for 4 weeks. Four groups of female ICR mice were used, i.e., AKT/YapS127A/Ctrl without gefitinib, *n* = 4; AKT/YapS127A/Ctrl with gefitinib, *n* = 4; AKT/YapS127A/*LAMC2* without gefitinib, *n* = 6; and AKT/YapS127A/*LAMC2* with gefitinib, *n* = 8, These mice were sacrificed 9 weeks post‐injection.

For each injection, the combined plasmids were diluted in 2 mL saline (0.9% NaCl), filtered through a 0.22 µm filter, and injected into the lateral tail vein of mice in 5–7 s. The detail plasmid combination and amount are listed in Table S6 (Supporting Information).

### Conditioned Medium Preparation

When the cultured cells reached ≈90% confluency, the medium was replaced with fresh serum‐free medium. Twelve hours later, the conditioned medium containing cell secretome was collected and centrifuged at 800 rpm for 5 min, to remove cell debris. The collected conditioned medium was either immediately used accordingly or stored at −80 °C to be used within 2 weeks.

### Cell Viability Assay, Colony Formation Assay, and Wound Healing Assay

Cell viability was detected using 3‐(4,5‐dimethylthiazol‐2‐yl)−2,5‐diphenyltetrazolium bromide (MTT, Cat# 298‐93‐1, Sangon Biotech) assay. For *LAMC2* knockdown phenotype experiment assay, RBE (1000 cells/well) or HUCCT1 (500 cells per well) cells were seeded in 96‐well plates and cultured for 6 days. Cell viability was measured each day. For EGFR TKIs treatment assay, RBE (3000 cells per well) or HUCCT1 (4000 cells per well) cells were seeded in 96‐well plates and exposed to EGFR TKIs at the indicated concentrations. After 24–72 h of incubation, cell viability was measured.

For colony formation assay, RBE (1000 cells per well) or HUCCT1 (500 cells per well) cells were seeded in 6‐cm dishes and cultured for 12 days. Colonies were fixed with methanol, stained with crystal violet, and counted.

For wound healing assay, RBE or HUCCT1 cells were seeded in 6‐well plates and infected with the corresponding shRNA virus, artificial would tracks were generated in confluent monolayer cells by scraping with a 20 µL pipette tip. After removal of the detached cells by gently washing with PBS, the cells were incubated with a fresh complete medium. Images were acquired from 6 different fields for each group at the initial time and the later indicated time points. The remaining wound was measured and compared.

### Protein Extraction and Western Blot

Cell pellets were collected and lysed in IP buffer (0.5% NP40, 50 mm tris pH 7.5, 150 mm NaCl, 2 mm EDTA). A conditioned medium (CM) was used to detect the secreted proteins. For western blot, total cell lysates or CM were separated by SDS‐PAGE Gel and transferred to PVDF membranes. The membranes were incubated with indicated primary antibodies and secondary antibodies conjugated to horseradish peroxidase. The substrate signals were detected by chemiluminescence (Cat# 4AW011‐1000, 4A Biotech). These antibodies were *LAMC2* Rabbit Polyclonal antibody (Cat# A1869, ABclonal), β‐actin Rabbit Monoclonal antibody (Cat# AC026, ABclonal), EGFR Rabbit Monoclonal antibody (Cat# 4267, Cell signaling technology), p‐EGFR Rabbit Monoclonal antibody (Tyr1068, Cat# 3777, Cell signaling technology), p‐Erk1/2 Rabbit Monoclonal antibody (Thr202/Tyr204, Cat# 4370, Cell signaling technology), HA‐tag Rabbit Monoclonal antibody (Cat# 3724, Cell signaling technology), flag‐tag Mouse Monoclonal antibody (Cat# F3165, Sigma–Aldrich), BiP Rabbit Monoclonal antibody (Cat# 3177, Cell signaling technology), HRP‐linked anti‐rabbit IgG antibody (Cat# 129 736, Jackson Immuno Research), and HRP‐linked anti‐mouse IgG antibody (Cat# 129 457, Jackson Immuno Research).

### Immunohistochemistry (IHC)

IHC was performed on formaldehyde‐fixed paraffin‐embedded (FFPE) tissues from iCCA and HCC patients. *LAMC2* Rabbit Polyclonal antibody (Cat# A1869, ABclonal), EGFR Rabbit Polyclonal antibody (Cat# HPA018530, Sigma–Aldrich), and 2‐step plus Poly‐HRP Anti‐Mouse/Rabbit IgG EnVision Detection System (PV‐8000, ZSGB‐BIO, China) was used. For each sample, the staining area was evaluated from 0 to 4 (0, 0–5%; 1, 5–25%; 2, 25–50%; 3, 50–75%; 4, >75%) and the intensities were graded from 0 to 3 (0, negative; 1, weak; 2, moderate; 3, strong). A final IHC score between 0 and 12 was achieved by multiplication of staining area and intensity as before.^[^ ^40^ ^]^

### Immunoprecipitation (IP), Mass Spectrometry, Tandem IP/Mass Spectrometry

For IP, total cells were lysed in IP buffer, cell lysates were incubated with anti‐Flag‐M2 magnetic beads (Cat# M8823, Sigma–Aldrich), Pierce anti‐HA magnetic beads (Cat# 88 836, Thermo Fisher Scientific) or Pierce protein A/G magnetic beads (Cat# 88 802, Thermo Fisher Scientific), or with indicated antibodies at 4 °C overnight. These antibodies were *LAMC2* Rabbit Polyclonal antibody (Cat# A1869, ABclonal) and BiP Rabbit Monoclonal antibody (Cat# 3177, Cell signaling technology). After washing, the immunoprecipitated proteins were subjected to immunoblotting.

For mass spectrometry, RBE cells transfected with siCtrl or si*LAMC2* #1,2 were collected by scraping. The biological triplicates were prepared. The cells were sent for mass spectrometry analysis in the institute.

For Tandem IP, RBE cells with overexpressed *LAMC2*‐flag‐HA were lysed in IP buffer and the cell lysates were incubated with anti‐Flag‐M2 magnetic beads at 4 °C overnight as the 1st IP. After washing, beads were incubated with 200 ng µL^−1^ flag peptide (ABclonal) at 4 °C for 2 h to compete and free *LAMC2*‐flag‐HA proteins. Then, the supernatants were collected and incubated with Pierce anti‐HA magnetic beads at 4 °C overnight as the 2nd IP. After washing, proteins were boiled off from beads in 1% SDS loading buffer. The Tandem IP products were divided by SDS‐PAGE gel for 0.8–1 cm and the gel was stained with coomassie brilliant blue G250. The stained gel was sliced out, distanced, and sent for mass spectrometry analysis in the institute.

### Immunofluorescence (IF)

Cells were seeded on coverslips, fixed with precooled methanol at −20 °C for 5 min, permeabilized with 0.1% Triton X‐100 for 10 min, and blocked with 3% BSA for 1 h. After being incubated with primary antibodies at room temperature for 1 h, cells were then further incubated with the corresponding secondary antibodies for 1 h at room temperature. Nuclei were stained with DAPI in the mounting reagent (Sangon Biotech). Confocal fluorescence images were captured using a Zeiss LSM 880 AiryScan laser microscope. These antibodies were *LAMC2* Rabbit Polyclonal antibody (Cat# A1869, ABclonal), EGFR Mouse Monoclonal antibody (Santa Cruz), and Alexa Fluor 594 Calreticulin Rabbit Monoclonal antibody (Cat# ab275343, Abcam).

### PNGase F Treatment Assay

The presence of glycans in EGFR was determined using peptide‐N‐glycosidase F (PNGase F). For treatment with PNGase F (Cat# P0708S, New England Biolabs), total cells were lysed in IP buffer and the cell lysates were incubated with Pierce anti‐HA magnetic beads at 4 °C overnight. After washing, the immunoprecipitated proteins and input proteins were pre‐denatured in Glycoprotein Denaturing Buffer at 100 °C for 10 min, and the denatured proteins were treated with PNGase F in a mixture with GlycoBuffer 2 (10X), 10% NP‐40 and deionized water at 37 °C for 1 h according to the manufacturer's instructions. The digested lysates were subjected to immunoblotting.

### Boncat Assay with L‐AHA, Followed with Click Chemistry Reaction

Boncat is an abbreviation of Bioorthogonal Non‐canonical Amino Acid Tagging. For this assay, Cells were cultured with high glucose, no glutamine, no methionine, and no cysteine DMEM (Cat# 21 013 024, Gibico) for 1 h, then labeled with 50 µm L‐Azidohomoalanine (L‐AHA, Cat# 900 892, Sigma–Aldrich) for 2 h for detecting endogenous proteins or 6 h for detecting exogenous proteins. Specifically, to detect the translation of EGFR^13Q^, 12 h of L‐AHA labeling was used. Total cells were lysed with lysis buffer (1%SDS, 50 mm tris pH 8.0) including protease inhibitors and Benzohase endonuclease (Cat# E1014, Sigma–Aldrich). The collected supernatants were then incubated with 100 µm Biotin‐alkyne (Cat# 764 213, Sigma–Aldrich) using Click‐iT Protein Reaction Buffer Kit (Cat# C10276, Thermo Fisher Scientific). After chemistry reactions, proteins were precipitated with methanol, chloroform, and deionized water, and dissolved by IP buffer afterward. The dissolved proteins were then incubated with Streptavidin‐agarose beads (Cat# 20 347, Thermo Fisher Scientific) at 4 °C for 4 h. After washing, the immunoprecipitated proteins were subjected to immunoblotting to examine the newly synthesized proteins.

### Statistical Analysis

Class comparison was used for screening for genes with significant alteration in iCCAs. Hierarchical clustering analysis was performed by the GENESIS software version 1.7.6 developed by Alexander Sturn (IBMT‐TUG, Graz, Austria). Statistical software R (version 4.2.0, https://www.r‐project.org) was used for Principal Component Analysis (PCA). The first and second principal components were taken to plot the dissimilarities among tumor/non‐tumor iCCA and HCC samples. Students’ *t*‐test, one‐way ANOVA, and two‐way ANOVA were used for statistical analysis of comparative data between groups using Prism V8 software (Graphpad Inc.). Spearman correlation analysis was performed to examine the correlation of *LAMC2* and EGFR. Kaplan–Meier survival analysis was used for statistical analysis of patient survival between groups in Graphpad Prism V8 software, and the statistical p‐value was generated by the Cox–Mantel log‐rank test. Gene set enrichment analysis (GSEA) was performed between different *LAMC2* expression levels of iCCA tumors in Cohort 1 and Cohort 5. All results were presented as mean ± SD or median or median with range unless otherwise indicated. All *p*‐values were 2‐sided, and the statistical significance was defined as a *p*‐value of less than 0.05.

### Study Approval

Formalin‐fixed paraffin‐embedded (FFPE) tumor tissues from intrahepatic cholangiocarcinoma patients and hepatocellular carcinoma patients being used in the study were from Shandong Cancer Hospital and Institute in China. The institutional review board of Shandong Cancer Hospital and Institute approved the use of these FFPE tissues and waived the requirement for informed consent (No.2023011005, L. Zhao). All mouse procedures were conducted under the guidelines and the institutional animal care protocol approved by the Experimental Animal Committee at Zhejiang University (ZJU20200014, J. Ji)

## Conflict of Interest

The authors declare no conflict of interest.

## Author Contributions

J.Z. and F.J. contributed equally to this work and are joint first authors. J.J., J.Z., and F.J. contributed to the conception and design of this work. J.Z., F.J., Y.T., and Y.Z. contributed to the development of methodology. J.Z., F.J., Y.T., Y.Z., J.L., and L.S. were involved in the data acquisition of this work. J.Z., X.Z., and J.J. contributed to the data analysis and interpretation including statistical analysis, biostatistics, computational analysis, etc. J.Z., F.J., S.R., X.Z., and J.J. were involved in the writing, review, and/or revision of this manuscript. L.Z., J.S., M.Y., X.H., J.J., B.Z., J.H., and X.Z. offered administrative, technical, and material support to this work. Study supervision was done by J.J. All authors have approved the submitted version.

## Supporting information

Supporting Information

## Data Availability

The mRNA profiling data of cohort 1 were available at GEO datasets of NCBI (GSE76297, https://www.ncbi.nlm.nih.gov/geo/query/acc.cgi?acc=GSE76297). The mRNA sequencing data of cohort 2 were available at the Cancer Genome Atlas (TCGA) portal (https://portal.gdc.cancer.gov). The mRNA profiling data of cohort 3 were available at GEO datasets (GSE26566, https://www.ncbi.nlm.nih.gov/geo/query/acc.cgi?acc=GSE26566). The mRNA transcriptome data and tumor proteome data of cohort 5 were obtained from Dr. Jia Fan (https://www.sciencedirect.com/science/article/pii/S1535610821006590?via%3Dihub).
