# Supplementary material for: Oncogenic Roles of Laminin Subunit Gamma‐2 in Intrahepatic Cholangiocarcinoma via Promoting EGFR Translation
Source: Adv Sci (Weinh). 2024 Mar 25;11(21):2309010. doi: 10.1002/advs.202309010 (PMC11151066; doi:10.1002/advs.202309010)
Supplement: Supplementary file 1 — Supporting Information [file ADVS-11-2309010-s001.pdf]

ADVANCED SCIENCE
Open Access

Supporting Information

for *Adv. Sci.*, DOI 10.1002/advs.202309010

Oncogenic Roles of Laminin Subunit Gamma-2 in Intrahepatic Cholangiocarcinoma via Promoting EGFR Translation

*Jianjuan Zhang, Fubo Ji, Yaqi Tan, Lei Zhao, Yongzhi Zhao, Jiaxin Liu, Liyuan Shao, Jiong Shi, Meihua Ye, Xianglei He, Jianping Jin, Bin Zhao, Jun Huang, Stephanie Roessler, Xin Zheng and Junfang Ji**

Supplementary data

Oncogenic roles of Laminin subunit gamma-2 in intrahepatic cholangiocarcinoma via promoting EGFR translation

Jianjuan Zhang[1,2,3,#], Fubo Ji[1,2,3,#], Yaqi Tan[1,2,3], Lei Zhao[4], Yongzhi Zhao[1,2,3], Jiaxin Liu[1,2,3], Liyuan Shao[2], Jiong Shi[5], Meihua Ye[6], Xianglei He[6], Jianping Jin[1,2,3], Bin Zhao[1,2,3], Jun Huang[1,2,3], Stephanie Roessler[7], Xin Zheng[8], Junfang Ji[1,2,3,*.]

[#]The authors wish it to be known that, in their opinion, the first two authors should be regarded as joint First Authors.

***To whom correspondence should be addressed:** Dr. Junfang Ji, The MOE Key Laboratory of Biosystems Homeostasis & Protection, Zhejiang Provincial Key Laboratory for Cancer Molecular Cell Biology, Life Sciences Institute, Zhejiang University, 866 Yuhangtang Road, Hangzhou, Zhejiang Province, China 310058, Tel: +86 571 88208956, Email: junfangji@zju.edu.cn

Contents of this Supplementary file

- **Supplementary Tables, n=6...................................... page s2-s9**
- **Supplementary Figure legends……………………….page s10-s13**
- **Supplementary Figures, n=14..………………………..page s14-s27**

Supplementary Tables

Table S1. Summary of Datasets and Clinical specimens used in this study.

Cohorts	Source	Cases	Datasets or Specimens
Cohort 1	GSE76297	91 iCCA cases; 62 HCC cases.	mRNA array (paired tumor & non-tumor tissues)
Cohort 2	TCGA	36 CCA cases (iCCA, n=31)	RNA sequencing (tumor, n=36; non-tumor, n=9)
Cohort 3	GSE26566	104 CCA cases (iCCA, n=68)	mRNA array (tumor, n=104; non-tumor, n=59; normal bile duct, n=6)
Cohort 4	Shandong cancer hospital	33 iCCA cases; 20 HCC cases.	FFPE tissues of iCCA cases, n=33; FFPE tissues of HCC cases, n=20.
Cohort 5	Ref (*Cancer Cell, 2022.*)	262 iCCA cases	RNA sequencing (tumor, n=255); Proteome (tumor, n=214).

Table S2. Clinical Characteristics of Cohort 4.

Clinical Variable		iCCA, n=33	HCC, n=20	p-value
Gender	Female	10	1	0.04[a]
	Male	21	17	
	Missing value	2	2	
Age-year	Median (range)	59(43-77)	53(31-72)	0.02[b]
ALT (U/L)	Normal (≤55)	23	16	0.29[a]
	Abnormal (>55)	8	2	
	Missing value	2	2	
AST (U/L)	Normal (≤48)	21	16	0.17[a]
	Abnormal (>48)	10	2	
	Missing value	2	2	
Albumin (g/L)	Normal (≥35)	24	18	0.04[a]
	Abnormal (<35)	7	0	
	Missing value	2	2	
AFP (ng/ml)	Normal (≤20)	17	10	0.32[a]
	Abnormal (>20)	6	8	
	Missing value	10	2	
CA19-9 (U/ml)	Normal (≤40)	7	/	/
	Abnormal (>40)	20	/	
	Missing value	6	/	
CEA (ng/ml)	Normal (≤5)	13	/	/
	Abnormal (>5)	13	/	
	Missing value	7	/	
BCLC stage	0-A	/	12	/
	B-D	/	6	
	Missing value	/	2	
TNM Stage	I-II	10	12	0.07[a]
	III-IV	18	6	
	Missing value	5	2	
Multi-nodules	No	0	16	<0.001[a]
	Yes	7	2	
	Missing value	26	2	
Tumor size	≤3 cm	4	9	0.04[a]
	>3 cm	20	9	
	Missing value	9	2	

a: Fisher’s exact test; b: Unpaired t-test

Table S3. Clinical Characteristics of LAMC2^{high} and LAMC2^{low} group in Cohort 4.

Clinical Variable	IHC staining score			*LAMC2* amplification		
	LAMC2^{high} Score ≥ 6 n=24	LAMC2^{low} Score < 6 n=9	p-value	LAMC2^{high} Copies ≥ 4 n=5	LAMC2^{low} Copies < 4 n=14	p-value
Gender						
Female	7	3	1.00[a]	2	5	1.00[a]
Male	15	6		3	9	
Missing value	2	0		0	0	
Age-year						
Median (range)	59 (43-77)	62 (45-69)	0.91[b]	53 (43-72)	64 (45-77)	0.14[b]
ALT (U/L)						
Normal (≤55)	16	7	1.00[a]	4	10	1.00[a]
Abnormal (>55)	6	2		1	4	
Missing value	2	0		0	0	
Albumin (g/L)						
Normal (≥35)	17	7	1.00[a]	3	12	0.27[a]
Abnormal (<35)	5	2		2	2	
Missing value	2	0		0	0	
AFP (ng/ml)						
Normal (≤20)	11	6	0.14[a]	3	6	0.50[a]
Abnormal (>20)	6	0		0	4	
Missing value	7	3		2	4	
CA19-9 (U/ml)						
Normal (≤40)	4	3	0.65[a]	0	5	0.28[a]
Abnormal (>40)	14	6		4	9	
Missing value	6	0		1	0	
CEA (ng/ml)						
Normal (≤5)	11	2	0.38[a]	1	7	0.28[a]
Abnormal (>5)	8	5		4	4	
Missing value	5	2		0	3	
TNM Stage						
I-II	7	3	1.00[a]	0	4	0.52[a]
III-IV	13	5		4	9	
Missing value	4	1		1	1	
Tumor size (cm)						
≤3	3	0	0.53[a]	0	1	1.00[a]
>3	14	7		5	10	
Missing value	7	2		0	3	
Overall survival-month						
Median	16.2	29.8	0.15[c]	9.2	28.2	0.14[c]
Range	1.8-36.9	3.5-35		3.2-18.4	1.8-35	

a: Fisher's exact test; b: Unpaired t-test; c: Log-rank test

Table S4. Clinical Characteristics of LAMC2^{high} and LAMC2^{low} group in Cohort 5.

Clinical Variable	LAMC2^{high} n=107	LAMC2^{low} n=107	p-value
Gender			
Female	47	47	1.00[a]
Male	60	60	
Age-year			
Median (range)	65 (39-86)	60 (28-83)	0.05[b]
ALT (U/L)			
Normal (≤55)	90	96	0.31[a]
Abnormal (>55)	17	11	
Albumin (g/L)			
Normal (≥35)	103	106	0.37[a]
Abnormal (<35)	4	1	
AFP (ng/ml)			
Normal (≤20)	100	92	0.11[a]
Abnormal (>20)	7	15	
CA19-9 (U/ml)			
Normal (≤40)	32	66	<0.001[a]
Abnormal (>40)	75	41	
CEA (ng/ml)			
Normal (≤5)	70	94	<0.001[a]
Abnormal (>5)	37	13	
TNM Stage			
I-II	64	75	0.15[a]
III-IV	43	32	
Tumor size (cm)			
≤3	16	16	1.00[a]
>3	91	91	
Intrahepatic metastasis			
No	65	78	0.08[a]
Yes	42	29	
Distal metastasis			
No	100	104	0.33[a]
Yes	7	3	
Overall survival-month			<0.001[c]
Median	16.4	23.9	
Range	1.4-52.7	2.4-60.2	

a: Fisher's exact test; b: Unpaired t-test; c: Log-rank test

Table S5. Primers and oligoes used in this study.

Primers	Sequence (5'-3')
	**TB Green real-time PCR**
LAMC2	F: GACAAACTGGTAATGGATTCCGC
	R: TTCTCTGTGCCGGTAAAAGCC
EGFR	F: TTGCCGCAAAGTGTGTAACG
	R: GTCACCCCTAAATGCCACCG
18S	F: GACTCAACACGGGAAACCTC
	R: AGCATGCCAGAGTCTCGTTC
LAMC2-mus	F: CAGACACGGGAGATTGCTACT
	R: CCACGTTCCCCAAAGGGAT
18S-mus	F: GCCGCTAGAGGTGAAATTCTT
	R: CGTCTTCGAACCTCCGACT
FOS	F: CACTCCAAGCGGAGACAGAC
	R: AGGTCATCAGGGATCTTGCAG
	**siRNA target sequence**
siLAMC2 #1	GGUUCUCUUAGUGCUCGAU
siLAMC2 #2	GCAGAAUACAGUGUCCAUA
siBiP #1	GCGUCGGCGUGUUCAAGAA
siBiP #2	CCAUGCAGUUGUUACUGUA
siPERK #1	GGAUAGUGACGAAAUGGAA
siPERK #2	GAUACAAGUUAUACAUCUA
	**Primers for vector construction**
pcDNA3.0-LAMC2-HA	F: CGGGATCCGCCACCATGCCTGCGCTCTGGCTG
	R: CCGCTCGAGCTGTTGCTCAAGAGCCTGGG
pcDNA3.0-N-LAMC2-HA	F: TGGTACCGAGCTCGGATCCGCCACCATGCCTGCGCTCTGGC
	R: GACGTCGTATGGGTACTCGAGGCTGAATGCTCCATGCTCACAG
pcDNA3.0-C-LAMC2-HA	F1: TGGTACCGAGCTCGGATCCGCCACCATGCCTGCGCTCTGGC
	R1: GCAAGCTGGACAGACTTCCCTCCTGGAGGTGG
	F2: GAGGGAAGTCTGTCCAGCTTGCTATAATCAAGTGAAGA
	R2: GACGTCGTATGGGTACTCGAGCTGTTGCTCAAGAGCCTGGG
pcDNA3.0-LAMC2$^{\Delta Dv}$-HA	F1: TGGTACCGAGCTCGGATCCGCCACCATGCCTGCGCTCTGGC
	R1: AGAGCTGCGGACTTCCCTCCTGGAGGTGGC
	F2: AGGGAAGTCCGCAGCTCTGCAGAATACAGTG
	R2: GACGTCGTATGGGTACTCGAGCTGTTGCTCAAGAGCCTGGGTAT
pcDNA3.0-LAMC2$^{\Delta Div}$-HA	F1: TGGTACCGAGCTCGGATCCGCCACCATGCCTGCGCTCTGGC
	R1: CCCAACAGGACATATACAGCAGCTGGCTGAATGCCC
	F2: TGTATATGTCCTGTTGGGTACAAGGGGCAATTC
	R2: GACGTCGTATGGGTACTCGAGCTGTTGCTCAAGAGCCTGGGTAT
pcDNA3.0-LAMC2$^{\Delta Diii}$-HA	F1: TGGTACCGAGCTCGGATCCGCCACCATGCCTGCGCTCTGGC
	R1: ATTATAGCAAGCTGGACACTGTTCAACCCAGGGTGC
	F2: CAGTGTCCAGCTTGCTATAATCAAGTGAAGATTCAG
	R2: GACGTCGTATGGGTACTCGAGCTGTTGCTCAAGAGCCTGGGTAT
pcDNA3.0-EGFR-HA	F: TGGTACCGAGCTCGGATCCGCCACCATGCGACCCTCCGGGACG
	R: GACGTCGTATGGGTACTCGAGTGCTCCAATAAATTCACTGCTTTGTGGC
pcDNA3.0-BiP-HA	F: CGGGATCCGCCACCATGAAGCTCTCCCTGGTGGC
	R: CCCTCGAGCTACAACTCATCTTTAGCGTAGTCTGGGACGTCG
pcDNA3.0-ERdj1-HA	F: CGGGATCCGCCACCATGACGGCTCCTTGCTCCC
	R: CCGCTCGAGGCTTTTAGCTTGTTTTTTCTTTTGGACC
pcDNA3.0-ERdj2-HA	F: CGGGATCCGCCACCATGGCCGGGCAGCAGTT
	R: CCGCTCGAGGTCATCATCTTCTTCTTCCTCCTCTTCTTC
p3xflag-cmv-14-EGFR	F: CCAAGCTTGCCACCATGCGACCCTCCGGGAC
	R: GCTCTAGATGCTCCAATAAATTCACTGCTTTGTGG
p3xflag-cmv-14-EGFR-extra	F: CCAAGCTTGCCACCATGCGACCCTCCGGGAC
	R: GCTCTAGAGGACGGGATCTTAGGCCCAT
p3xflag-cmv-14-EGFR-cyto	F: CCAAGCTTGCCACCATGCGAAGGCGCCACATCGTTC
	R: GCTCTAGATGCTCCAATAAATTCACTGCTTTGTGG

p3xflag-cmv-14-$EGFR^{13Q}$	F1: TGAACCGTCAGAATTAAGCTTGCCACCATGCGACCCTCCGGGAC<br>R1: AAGCTGTATTTGCCCTCGGGGTTCACATC<br>F2: CCCCGAGGGCAAATACAGCTTTGGTGCCACCTG<br>R2: AGACCTGGCCCAGTGCATCCGTAGGTG<br>F3: ATGCACTGGGCCAGGTCTTGAAGGCTGTCC<br>R3: CCGGGATCCTCTAGATGCTCCAATAAATTCACTGCTTTGTGG
pcDNA3.0-BiP-flag	F1: AAGCTTGGTACCGAGCTCGGATCCGCCACCATGAAGCTCTCCCTGGTGGC<br>R1: CTTTGTAGTCTTCTGCTGTATCCTCTTCACCAGTTG<br>F2: CAGCAGAAGACTACAAAGACCATGACGGTGAT<br>R2: CCCTCTAGATGCATGCTCGAGCTACAACTCATCTTTCTTGTCATCGTCATCC
pcDNA3.0-BiP-Δ19-124-flag	F1: AAGCTTGGTACCGAGCTCGGATCCGCCACCATGAAGCTCTCCCTGGTGGC<br>R1: GAATGTATGGTTTGGCCCGCGCCG<br>F2: CGGGCCAAACCATACATTCAAGTTGATATTGGAGGTGG<br>R2: CCCTCTAGATGCATGCTCGAGCTACAACTCATCTTTCTTGTCATCGTCATCC
pcDNA3.0-BiP-ΔNBD-flag	F1: AAGCTTGGTACCGAGCTCGGATCCGCCACCATGAAGCTCTCCCTGGTGGC<br>R1: GCACAGCTCTATTGTCAGTTTTCTTTTCAACCACCTTGAACGG<br>F2: GAAAACTGACAATAGAGCTGTGCAGAAACTCC<br>R2: CCCTCTAGATGCATGCTCGAGCTACAACTCATCTTTCTTGTCATCGTCATCC
pcDNA3.0-BiP-Δ281-419-flag	F1: AAGCTTGGTACCGAGCTCGGATCCGCCACCATGAAGCTCTCCCTGGTGGC<br>R1: AGGGGACATTTCCTGACATCTTTGCCCGTC<br>F2: GTCAGGAAATGTCCCCTTACACTTGGTATTGAAACTG<br>R2: CCCTCTAGATGCATGCTCGAGCTACAACTCATCTTTCTTGTCATCGTCATCC
pcDNA3.0-BiP-ΔSBD-flag	F1: AAGCTTGGTACCGAGCTCGGATCCGCCACCATGAAGCTCTCCCTGGTGGC<br>R1: CTATCTCAAATACATCAAGCAGTACCAGGTCACC<br>F2: CCTGGTACTGCTTGATGTATTTGAGATAGATGTGAATGGTATTCTTCGAGTG<br>R2: CCCTCTAGATGCATGCTCGAGCTACAACTCATCTTTCTTGTCATCGTCATCC
pcDNA3.0-BiP-Δ501-650-flag	F1: AAGCTTGGTACCGAGCTCGGATCCGCCACCATGAAGCTCTCCCTGGTGGC<br>R1: TTGTAGTCGGTGACTTCAATCTGTGGGACCC<br>F2: CACAGATTGAAGTCACCGACTACAAAGACCATGACGGTGATT<br>R2: CCCTCTAGATGCATGCTCGAGCTACAACTCATCTTTCTTGTCATCGTCATCC
pcDNA3.0-LAMC2-flag-HA	F: TGGTACCGAGCTCGGATCCGCCACCATGCCTGCGCTCTGGCT<br>R: GACGTCGTATGGGTACTCGAGCTTGTCATCGTCATCCTTGTAGTCGAT
pcDNA3.0-LAMC2-V-flag-HA	F1: TGGTACCGAGCTCGGATCCGCCACCATGCCTGCGCTCTGGCT<br>R1: CTTGTCATCGTCATCCTTGTAGTCGACTTCCCTCCTGGAGGTGGC<br>F2: GACTACAAGGATGACGATGACAAGTGTGATTGCAATGGGAAGTCCAGG<br>R2: GACGTCGTATGGGTACTCGAGCTGTTGCTCAAGAGCCTGGGTAT
pcDNA3.0-LAMC2-III-flag-HA	F1: TGGTACCGAGCTCGGATCCGCCACCATGCCTGCGCTCTGGCT<br>R1: CTTGTCATCGTCATCCTTGTAGTCTGGCTTGCAGCTGCGGG<br>F2: GACTACAAGGATGACGATGACAAGTGTCCCTGTCATAACGGGTTCAG<br>R2: GACGTCGTATGGGTACTCGAGCTGTTGCTCAAGAGCCTGGGTAT
pLKO.1-shLAMC2 #1	F: CCGGGCCCTGCAATTGTAACTCCAACTCGAGTTGGAGTTACAATTGCAGGGCTTTTTG<br>R: AATTCAAAAAGCCCTGCAATTGTAACTCCAACTCGAGTTGGAGTTACAATTGCAGGGC
pLKO.1-shLAMC2 #2	F: CCGGGCTCACCAAGACTTACACATTCTCGAGAATGTGTAAGTCTTGGTGAGCTTTTTG<br>R: AATTCAAAAAGCTCACCAAGACTTACACATTCTCGAGAATGTGTAAGTCTTGGTGAGC
pLKO.1-sh-mLAMC2 #1	F: CCGGGCTACGTACGGAGAATATAGTCTCGAGACTATATTCTCCGTACGTAGCTTTTTG<br>R: AATTCAAAAAGCTACGTACGGAGAATATAGTCTCGAGACTATATTCTCCGTACGTAGCTTTTTG
pLKO.1-sh-mLAMC2 #2	F: CCGGGCCTCAACTGCAATGACAATACTCGAGTATTGTCATTGCAGTTGAGGCTTTTTG<br>R: AATTCAAAAAGCCTCAACTGCAATGACAATACTCGAGTATTGTCATTGCAGTTGAGGCTTTTTG
pT3-EF1α-myr-AKT-sh-mLAMC2 #1	F1: CCTTAATTAAGAGGGCCTATTTCCCATGATTCCTT<br>R1: GCTCTAGAATGAATACTGCCATTTGTCTCGAGGTC<br>F2: AGCAGGCAGAAGTATGCAAAGC<br>R2: GCTCTAGACAATTCCCACTCCTTTCAAGACCTAGAAG
pT3-EF1α-myr-AKT-sh-mLAMC2 #2	F1: CCTTAATTAAGAGGGCCTATTTCCCATGATTCCTT<br>R1: GCTCTAGAATGAATACTGCCATTTGTCTCGAGGTC<br>F2: AGCAGGCAGAAGTATGCAAAGC<br>R2: GCTCTAGACAATTCCCACTCCTTTCAAGACCTAGAAG
	F: GTACAAAAAAGCAGGCACGCCACATGCCTGCGCTCTGGC

pT3-EF1α-C-LAMC2	R: CCACAACTAGAATGCATCACTGTTGCTCAAGAGCCTGG
pT3-EF1α-N-LAMC2	F: GTACAAAAAAGCAGGCACGCCACATGCCTGCGCTCTGGC<br>R: CCACAACTAGAATGCATCAGCTGAATGCTCCATGCTCA
pT3-EF1α-$EGFR^{L858R}$	F: GTACAAAAAAGCAGGCACATGCGACCCTCCGGGAC<br>R: CCACAACTAGAATGCATCATGCTCCAATAAATTCACTGCTTTGTG

Table S6. The detailed usage of plasmids for HDTV mouse models.

Groups	Plasmids (μg)
pT3-EF1α-myr-AKT, pT3-EF1α-YapS127A, pCMV/SB	20, 30, 2
pT3-EF1α-myr-AKT, pT3-EF1α-NICD, pCMV/SB	4, 20, 1
pT3-EF1α-Myc, pCMV/SB	4, 0.16
pT3-EF1α-myr-AKT, NRasV12/pT2-CAGGS, pCMV/SB	4, 4, 0.32
pT3-EF1α-myr-AKT-shCtrl, pT3-EF1α-YapS127A, pCMV/SB	20, 30, 2
pT3-EF1α-myr-AKT-shLAMC2#m1, pT3-EF1α-YapS127A, pCMV/SB	20, 30, 2
pT3-EF1α-myr-AKT-shLAMC2#m2, pT3-EF1α-YapS127A, pCMV/SB	20, 30, 2
pT3-EF1α-myr-AKT-shCtrl, pT3-EF1α-NICD, pCMV/SB	4, 20, 1
pT3-EF1α-myr-AKT-shLAMC2#m1, pT3-EF1α-NICD, pCMV/SB	4, 20, 1
pT3-EF1α-myr-AKT-shLAMC2#m2, pT3-EF1α-NICD, pCMV/SB	4, 20, 1
pT3-EF1α-myr-AKT-shCtrl, pT3-EF1α-YapS127A, pT3-EF1α-CT, pCMV/SB	20, 30, 30, 3.2
pT3-EF1α-myr-AKT-shLAMC2#m1, pT3-EF1α-YapS127A, pT3-EF1α-CT, pCMV/SB	20, 30, 30, 3.2
pT3-EF1α-myr-AKT-shLAMC2#m1, pT3-EF1α-YapS127A, pT3-EF1α-LAMC2, pCMV/SB	20, 30, 30, 3.2
pT3-EF1α-myr-AKT-shLAMC2#m1, pT3-EF1α-YapS127A, pT3-EF1α-$EGFR^{L858R}$, pCMV/SB	20, 30, 30, 3.2
pT3-EF1α-myr-AKT-shCtrl, pT3-EF1α-YapS127A, pT3-EF1α-CT, pCMV/SB	20, 30, 30, 3.2
pT3-EF1α-myr-AKT-shLAMC2#m1, pT3-EF1α-YapS127A, pT3-EF1α-CT, pCMV/SB	20, 30, 30, 3.2
pT3-EF1α-myr-AKT-shLAMC2#m1, pT3-EF1α-YapS127A, pT3-EF1α-C-LAMC2, pCMV/SB	20, 30, 30, 3.2
pT3-EF1α-myr-AKT-shLAMC2#m1, pT3-EF1α-YapS127A, pT3-EF1α-N-LAMC2, pCMV/SB	20, 30, 30, 3.2
pT3-EF1α-myr-AKT, pT3-EF1α-YapS127A, pT3-EF1α-CT, pCMV/SB	6.7, 10, 20, 1.5
pT3-EF1α-myr-AKT, pT3-EF1α-YapS127A, pT3-EF1α-LAMC2, pCMV/SB	6.7, 10, 20, 1.5

Supplementary Figure legends：

Figure S1. A high expression of LAMC2 in iCCA tumor cells. (A) Principal component analysis of iCCA tumors and non-tumors, HCC tumors and non-tumors in Cohort 1. **(B)** Single-cell RNA-sequencing results of LAMC2 in major cell types and tumor cell types from liver cancer. T, tumor; NT, non-tumor.

Figure S2. Knockdown LAMC2 blocked iCCA cell proliferation, colony formation and migration. (A, B) LAMC2 shRNA knockdown efficiency in protein level (A) and mRNA level (B) in RBE and HUCCT1 cells. **(C, D)** Relative cell viability (C) and colony formation (D) were examined in RBE and HUCCT1 cells infected with shCtrl and shLAMC2 lentivirus. **(E, F)** Cell migration was examined in RBE (E) and HUCCT1 (F) cells infected with shCtrl and shLAMC2 lentivirus by wounding healing assay. The remaining wound was measured at the indicated time. **(G)** Mouse LAMC2 shRNA knockdown efficiency in mRNA level was detected in mouse primary hepatocyte H2.35 cell line. (C, E, F) Two-way ANOVA was used. (D) Student's t-test was used.

Figure S3. ~150kD full-length LAMC2 was the major form in iCCA cells. (A) LAMC2 expression was detected in cell lysate and conditioned medium of RBE and HUCCT1 cells. **(B)** LAMC2 major form detection. Top: schematic diagram of LAMC2 vector. Bottom: RBE and HUCCT1 cells were transfected with indicated vector LAMC2-flag-HA, collected cell lysate and conditioned medium for western blot analysis. **(C)** Schematic diagrams of LAMC2 protein and reported cleaved LAMC2 fragments. **(D)** LAMC2 major form detection. Top: schematic diagram of LAMC2 vectors. Bottom: RBE and HUCCT1 cells were transfected with indicated vectors LAMC2-V-flag-HA or LAMC2-III-flag-HA, collected cell lysate and conditioned medium for western blot analysis. **(E)** LAMC2 expression detection in cell lysate being collected with scraping method or trypsin method.

Figure S4. Secreted extracellular LAMC2 had no effect on iCCA cell proliferation and colony formation. (A) RBE cells transfected with siLAMC2 + Ctrl vector (indicating LAMC2-low status), or siCtrl + LAMC2-flag-HA vector (indicating LAMC2-high status). Conditioned medium was collected according to the detailed methods in our manuscript. LAMC2 expression in cell lysate and conditioned medium were detected by western blot. The Coomassie blue staining indicated the equal loading of conditioned medium. **(B)** Relative cell viability and colony formation were examined in LAMC2-high and LAMC2-low RBE cells. **(C)** RBE cells were incubated with LAMC2-low or LAMC2-high conditioned medium, and then relative cell viability and colony formation were examined. Two-way ANOVA analysis was used for cell viability assay. Student's t-test were used for colony formation assay. NS, not significant.

Figure S5. Mass spectrometry results in RBE cells with LAMC2 silencing. (A) The flow chart of sample preparation for mass spectrometry. **(B)** Western blot validation of LAMC2-silencing in RBE cells. **(C)** GSEA analysis with significantly altered proteins ($P < 0.05$) between siCtrl and siLAMC2 samples. Among the top 20 enriched signatures, there were two EGF-related signatures, whose enrichment plotted images were shown. **(D)** EGFR protein intensities in mass spectrometry data were compared between siCtrl RBE cells and siLAMC2 RBE cells. The cluster analysis of siCtrl and siLAMC2 RBE cells based on 6 molecules in an EGF/EGFR signaling gene set.

Figure S6. Effects of EGF/EGFR on LAMC2 expression and effects of secreted LAMC2 on EGFR signaling. (A) Spearman correlation analysis of LAMC2 protein and EGFR protein in iCCA tumors from Cohort 5. **(B)** LAMC2 mRNA expression was detected in RBE and HUCCT1 cells transfected with EGFR-flag. **(C)** RBE and HUCCT1 cells starved 12h and stimulated with or without EGF (100 ng/ml) for 24h. LAMC2 and FOS mRNA expression were detected. **(D)** LAMC2-low CM and LAMC2-high CM were collected from RBE cells transfected with LAMC2 siRNA + Ctrl vector or control siRNA + LAMC2-flag-HA vector. LAMC2 protein was detected in cell lysate and CM by western blot (left). The Coomassie blue staining indicated the equal loading of CM (right). CM, conditioned medium. **(E)** RBE cells were incubated with LAMC2-low CM or LAMC2-high CM overnight, then stimulated with or without EGF (100 ng/ml) for 30min. Indicated proteins were detected by western blot analysis.

Figure S7. LAMC2 did not affect EGFR mRNA expression and its protein stability. (A) EGFR mRNA expression was detected in RBE and HUCCT1 cells transfected with LAMC2 siRNAs. **(B)** EGFR mRNA expression was detected in RBE and HUCCT1 cells transfected with LAMC2-HA. **(C)** After knocking down LAMC2 with siRNAs, RBE and HUCCT1 cells were treated with cycloheximide (CHX) (20 μg/ml) at indicated intervals and protein stability of EGFR was detected by western blot. **(D)** HUCCT1 cells were co-transfected with LAMC2-HA and EGFR-flag, then treated with CHX at indicated intervals. Cell lysates were collected, and EGFR was detected by western blot. **(E)** After knocking down LAMC2 with shRNAs, HUCCT1 cells were treated with CHX (20 μg/ml) at indicated intervals and protein stability of EGFR was detected by western blot.

Figure S8. LAMC2 interacted with an undersized EGFR in several non-iCCA cells and Boncat assay was performed. (A) LAMC2 IHC staining results from 21 cancer types in Human Protein Atlas database. **(B)** Endogenous IP assay with anti-LAMC2 antibody in five different non-iCCA cells. **(C)** Boncat assay in five different non-iCCA cell lines with LAMC2 silencing. L-

AHA were applied to treat cells for 2 hours.

Figure S9. LAMC2 did not regulate the degradation of nascent EGFR. (A) RBE and HUCCT1 cells were treated with or without tunicamycin (0.5 μg/ml) for 12h, and follwed by CHX (20 μg/ml), MG132 (20 μM) or CB-5083 (2 μM) treatment for 12h. The indicated proteins were detected by western blot. **(B)** RBE and HUCCT1 cells were transfected with $EGFR^{13Q}$-flag. 36 hours after transfection, cells were treated with CHX (20 μg/ml), Bafilomycin A1 (100 nM) or CB-5083 (2 μM) for 12h. Cell lysates were collected for western blot detection. **(C)** RBE and HUCCT1 cells were transfected with EGFR-flag. 36 hours after transfection, cells were treated with CHX (20 μg/ml), Bafilomycin A1 (100 nM) or CB-5083 (2 μM) for 12h. Cell lysates were collected for western blot detection. **(D)** HUCCT1 cells were transfected with $EGFR^{13Q}$-flag vector and infected with shLAMC2 virus. 36 hours later, cells were treated with CHX (20 μg/ml) at indicated intervals and protein stability of $EGFR^{13Q}$-flag was detected by western blot. **(E)** HUCCT1 cells were co-transfected with LAMC2-HA and $EGFR^{13Q}$-flag. 36 hours later, cells were treated with CHX (20 μg/ml) at indicated intervals and protein stability of $EGFR^{13Q}$-flag was detected by western blot.

Figure S10. EGFR extracellular domain interacted with BiP. 293T, RBE and HUCCT1 cells were co-transfected with BiP-HA and different EGFR-flag vectors treated with tunicamycin (0.5 μg/ml). IP was performed with anti-flag beads.

Figure S11. LAMC2 C-terminus together with BiP SBD were important for regulating EGFR translation. (A) Boncat assay was performed in RBE and HUCCT1 cells co-transfected with BiP-flag/$EGFR^{13Q}$-flag and an intact LAMC2, or N-LAMC2 (disabling the pocket formation). Cells were treated with L-AHA for 12 hours. **(B)** Boncat assay was performed in RBE and HUCCT1 cells co-transfected with LAMC2-HA /$EGFR^{13Q}$-flag and an intact BiP, or $BiP^{\Delta SBD}$ and $BiP^{\Delta 501\text{-}650}$ (disabling the pocket formation). Cells were treated with L-AHA for 12 hours.

Figure S12. High LAMC2 level was related to poor prognosis in iCCA patients. (A) LAMC2 level in iCCA tumors of Cohort 5 and a median cut-off of LAMC2. **(B)** Kaplan-Meier survival analysis of iCCA patients in Cohort 5 based on a tertial cut-off of LAMC2. **(C)** Kaplan-Meier survival analysis of iCCA patients in Cohort 5 based on a quartile cut-off of LAMC2.

Figure S13. BiP promoted EGFR protein translation via its NBD region and ERdj proteins. (A)

Boncat assay in cells co-transfected with EGFR-flag and an intact BiP, or BiP$^{\Delta NBD}$. **(B)** Boncat assay in cells co-transfected with EGFR-flag and BiP-flag, and ERdj1-HA. **(C)** Boncat assay in cells co-transfected with EGFR-flag and BiP-flag, and ERdj2-HA. **(D)** Boncat assay in cells transfected with EGFR-flag with or without silencing BiP, together with silencing PERK. L-AHA were applied to treat cells for 6 hours.

Figure S14. BiP expression, survival analysis and relationship with EGFR signaling activation. **(A)** HSPA5 (gene name for BiP) expression levels in Cohorts 1-3. Unpaired Student's t-test was used for Cohort 1 and Cohort 3. Non-parametric t-test was used for Cohort 2. NS, not significant; T, tumor; NT, non-tumor. **(B)** Kaplan-Meier survival analysis of iCCA patients from Cohort 5 based on BiP protein level (Left panel, the median cut-off; Middle panel, the tertial cut-off; Right panel, the quartile cut-off). **(C)** The enrichment analysis of HSPA5-high patients in EGFR signaling activation and non-activation groups subclassified by an EGF/EGFR signaling gene set in Cohort 1 and Cohort 5. The cut off of HSPA5-high and HSPA5-low was based on the median cut-off of HSPA5 in tumor tissues from each cohort.

Supplementary Figures

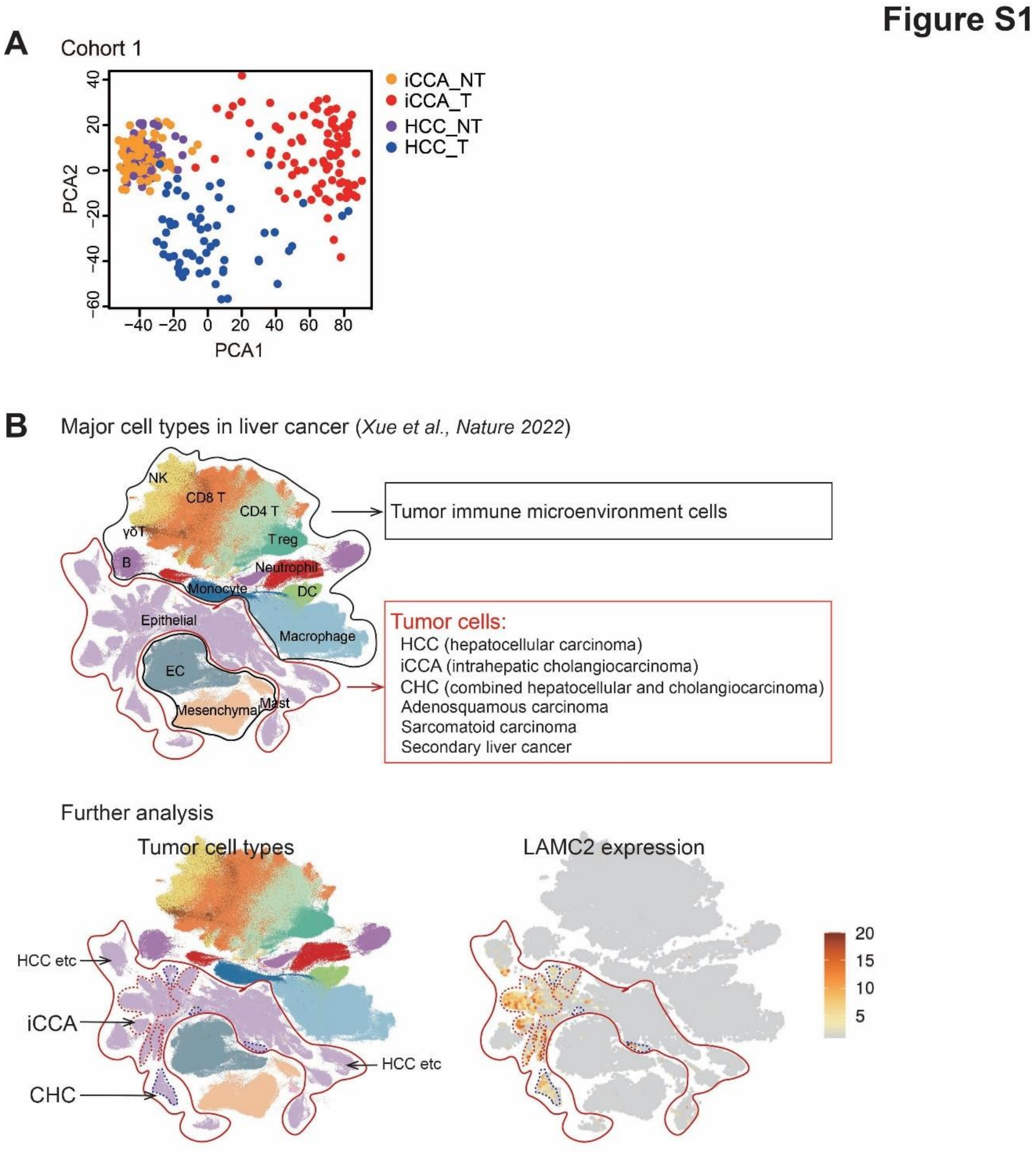

Figure S2

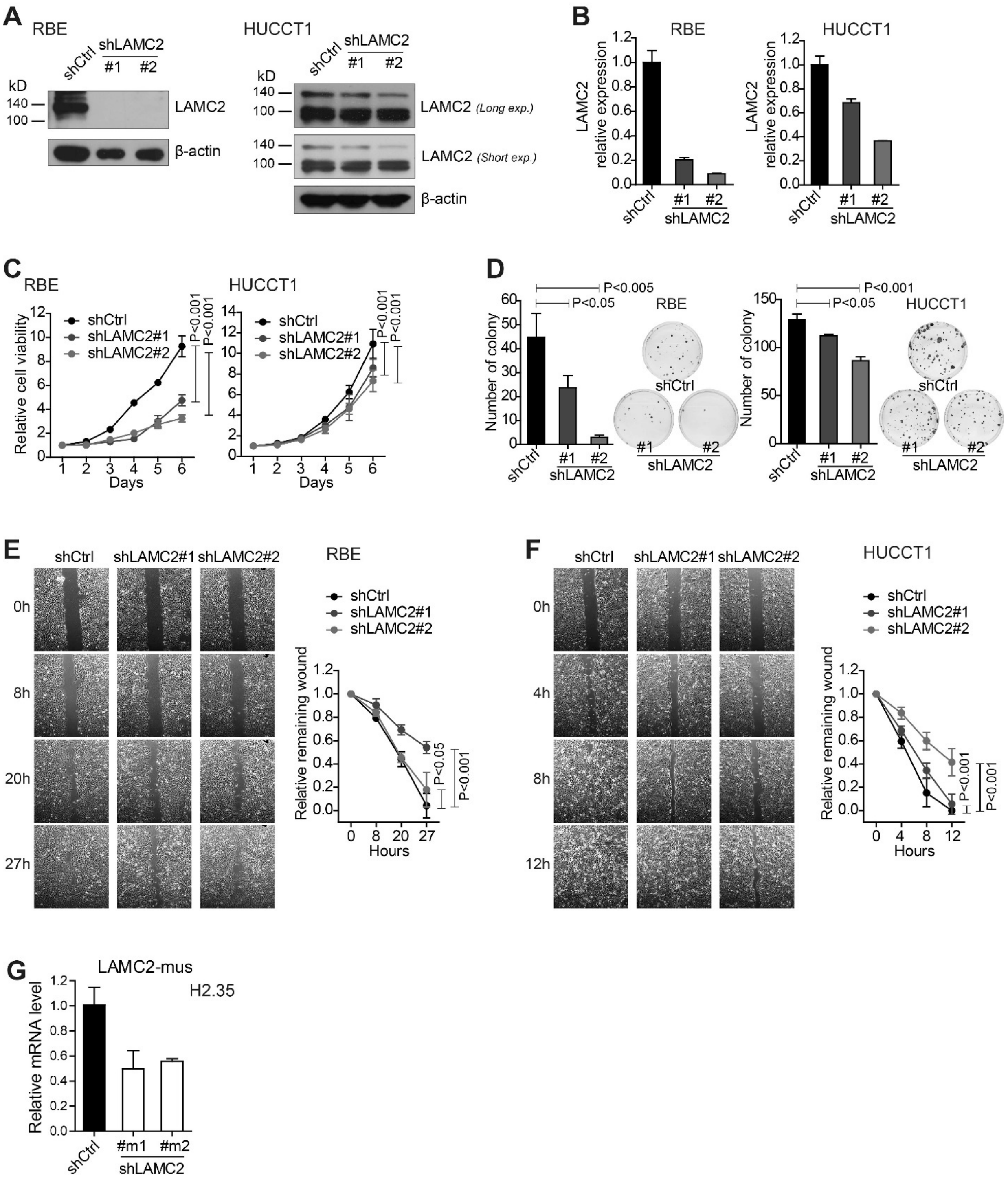

A
RBE
shCtrl
shLAMC2
#1
#2
kD
140
100
LAMC2
β-actin
HUCCT1
LAMC2 (Long exp.)
LAMC2 (Short exp.)
B
RBE
HUCCT1
LAMC2 relative expression
shCtrl
#1
#2
shLAMC2
C
RBE
HUCCT1
Relative cell viability
shCtrl
shLAMC2#1
shLAMC2#2
Days
P<0.001
P<0.001
D
P<0.005
P<0.05
P<0.001
Number of colony
RBE
HUCCT1
E
RBE
shCtrl
shLAMC2#1
shLAMC2#2
0h
8h
20h
27h
Relative remaining wound
Hours
P<0.05
P<0.001
F
HUCCT1
0h
4h
8h
12h
G
LAMC2-mus
H2.35
Relative mRNA level
#m1
#m2

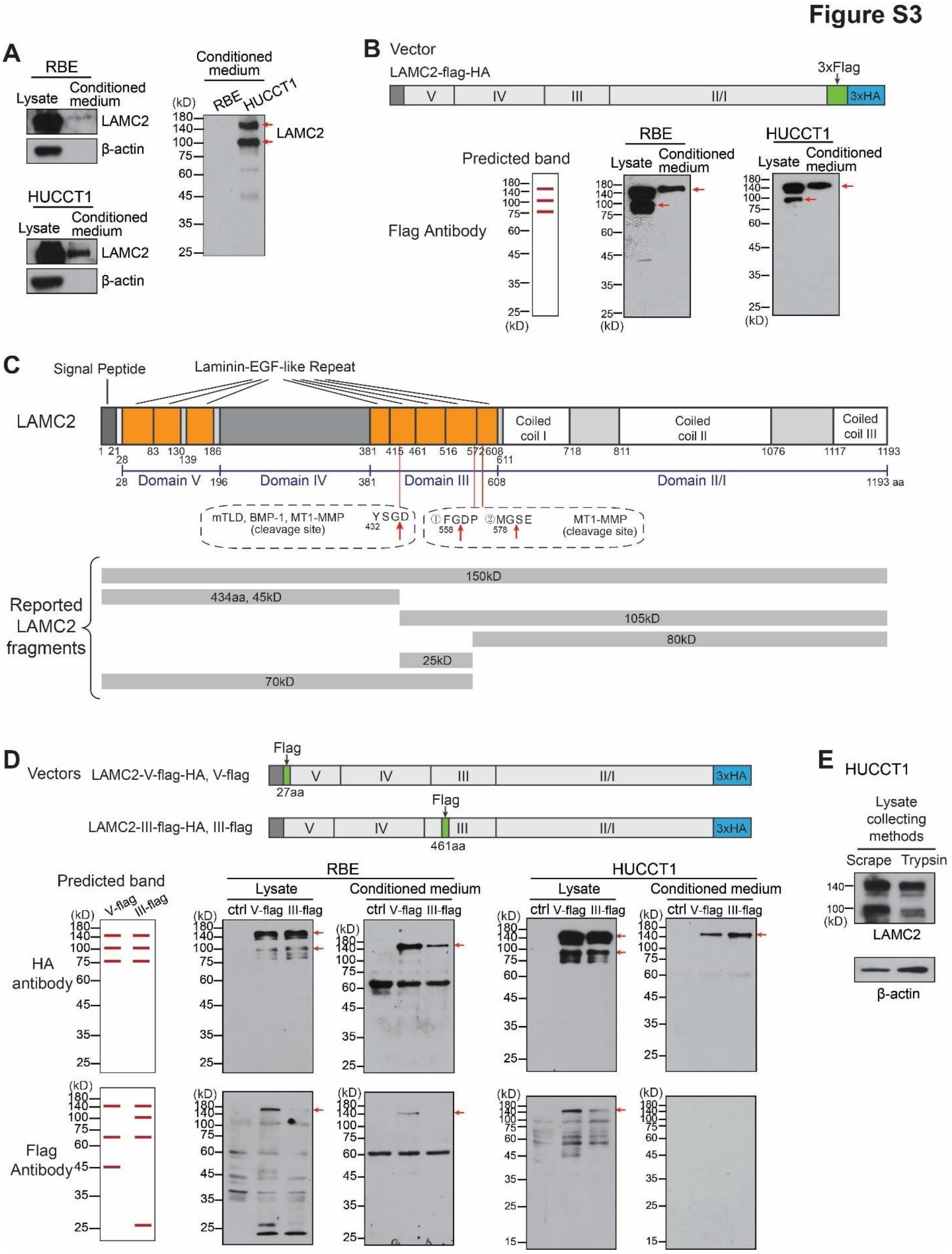
Figure S3
A
RBE
Lysate
Conditioned medium
LAMC2
β-actin
HUCCT1
Conditioned medium
RBE
HUCCT1
(kD)
B
Vector
LAMC2-flag-HA
3xFlag
V
IV
III
II/I
3xHA
Predicted band
Flag Antibody
C
Signal Peptide
Laminin-EGF-like Repeat
Coiled coil I
Coiled coil II
Coiled coil III
Domain V
Domain IV
Domain III
Domain II/I
mTLD, BMP-1, MT1-MMP (cleavage site)
YSGD
① FGDP ② MGSE
MT1-MMP (cleavage site)
Reported LAMC2 fragments
150kD
434aa, 45kD
105kD
80kD
25kD
70kD
D
Vectors
LAMC2-V-flag-HA, V-flag
Flag
27aa
LAMC2-III-flag-HA, III-flag
461aa
Predicted band
V-flag
III-flag
HA antibody
Flag Antibody
RBE
HUCCT1
Lysate
Conditioned medium
ctrl
E
HUCCT1
Lysate collecting methods
Scrape
Trypsin
LAMC2
β-actin

Figure S4

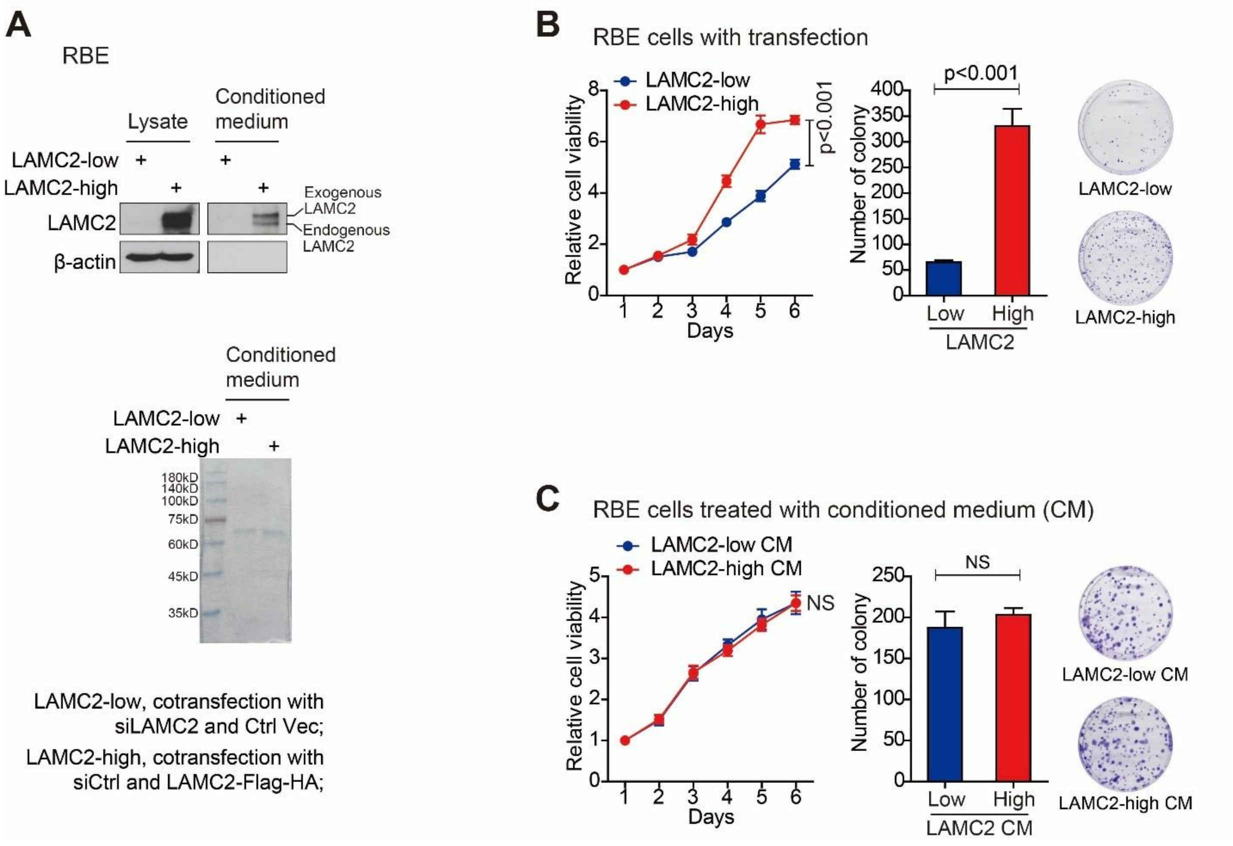
A
RBE
Lysate
Conditioned medium
LAMC2-low
LAMC2-high
LAMC2
β-actin
Exogenous LAMC2
Endogenous LAMC2
Conditioned medium
LAMC2-low
LAMC2-high
180kD
140kD
100kD
75kD
60kD
45kD
35kD
LAMC2-low, cotransfection with siLAMC2 and Ctrl Vec;
LAMC2-high, cotransfection with siCtrl and LAMC2-Flag-HA;
B
RBE cells with transfection
LAMC2-low
LAMC2-high
Relative cell viability
Days
p<0.001
Number of colony
Low
High
LAMC2
LAMC2-low
LAMC2-high
C
RBE cells treated with conditioned medium (CM)
LAMC2-low CM
LAMC2-high CM
NS
Relative cell viability
Days
Number of colony
Low
High
LAMC2 CM
LAMC2-low CM
LAMC2-high CM

Figure S5

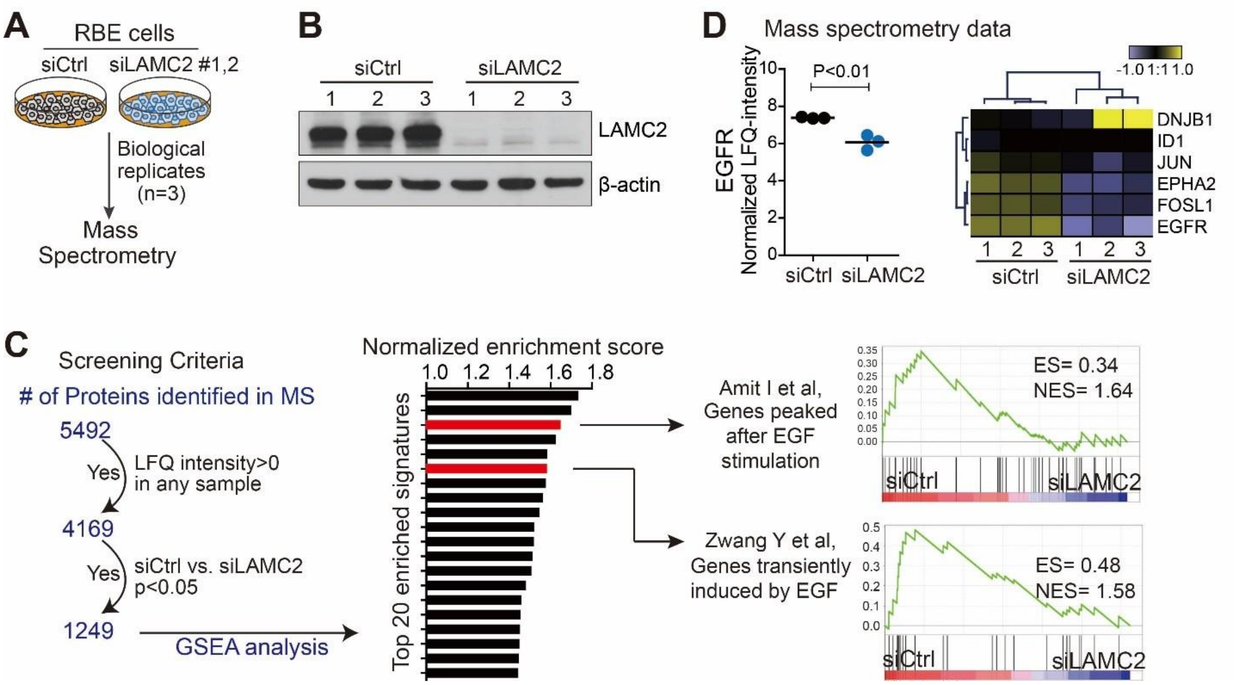
A
RBE cells
siCtrl
siLAMC2 #1,2
Biological replicates (n=3)
Mass Spectrometry
B
siCtrl
siLAMC2
1 2 3 1 2 3
LAMC2
β-actin
D
Mass spectrometry data
EGFR Normalized LFQ-intensity
P<0.01
siCtrl
siLAMC2
-1.0 1:1 1.0
DNJB1
ID1
JUN
EPHA2
FOSL1
EGFR
1 2 3 1 2 3
siCtrl
siLAMC2
C
Screening Criteria
# of Proteins identified in MS
5492
Yes
LFQ intensity>0 in any sample
4169
Yes
siCtrl vs. siLAMC2 p<0.05
1249
GSEA analysis
Normalized enrichment score
1.0 1.2 1.4 1.6 1.8
Top 20 enriched signatures
Amit I et al, Genes peaked after EGF stimulation
ES= 0.34
NES= 1.64
siCtrl
siLAMC2
Zwang Y et al, Genes transiently induced by EGF
ES= 0.48
NES= 1.58
siCtrl
siLAMC2

Figure S6

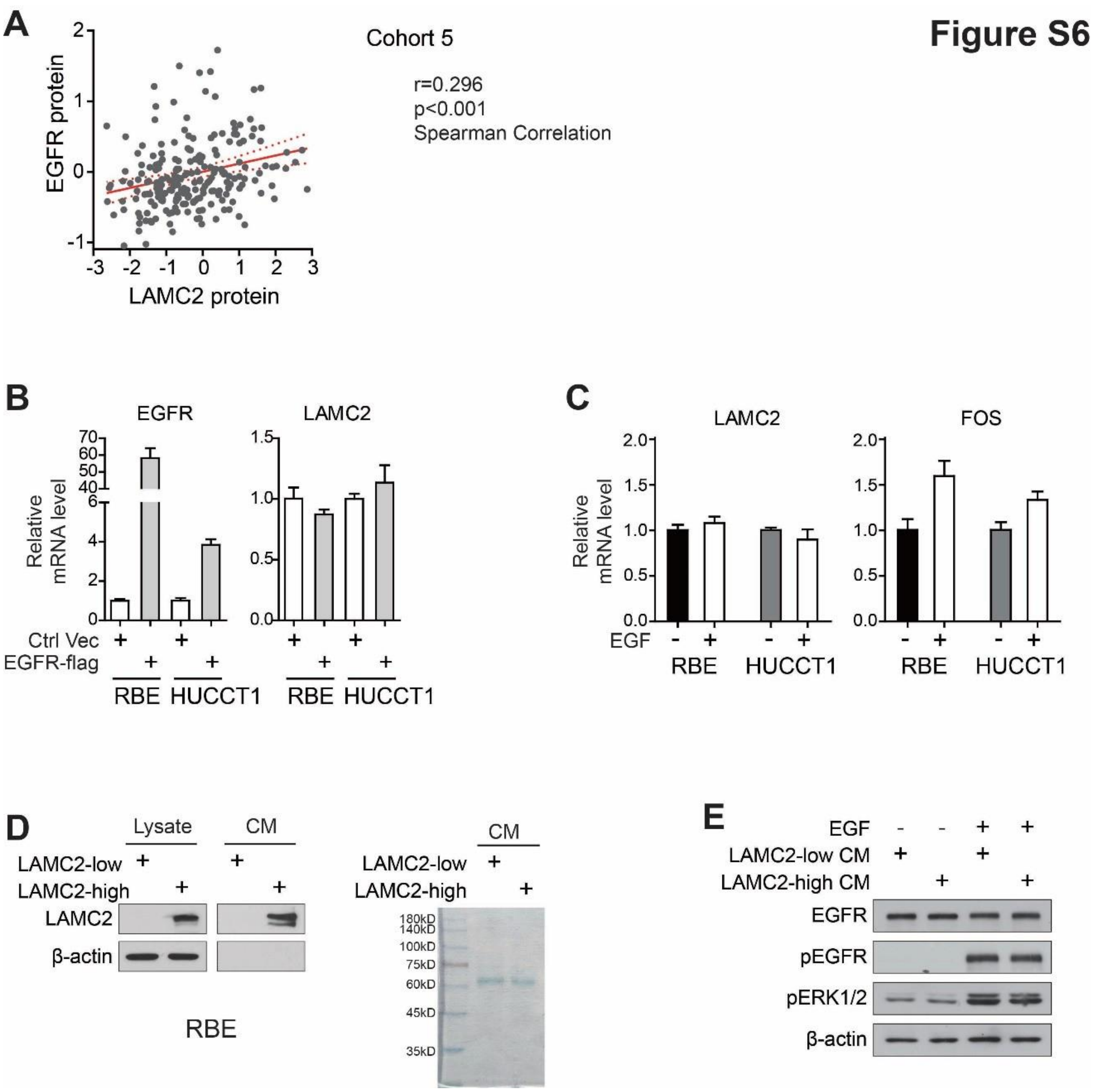
A
Cohort 5
r=0.296
p<0.001
Spearman Correlation
EGFR protein
LAMC2 protein
B
EGFR
LAMC2
Relative mRNA level
Ctrl Vec
EGFR-flag
RBE
HUCCT1
C
LAMC2
FOS
Relative mRNA level
EGF
RBE
HUCCT1
D
Lysate
CM
LAMC2-low
LAMC2-high
LAMC2
β-actin
RBE
180kD
140kD
100kD
75kD
60kD
45kD
35kD
E
EGF
LAMC2-low CM
LAMC2-high CM
EGFR
pEGFR
pERK1/2
β-actin

Figure S7

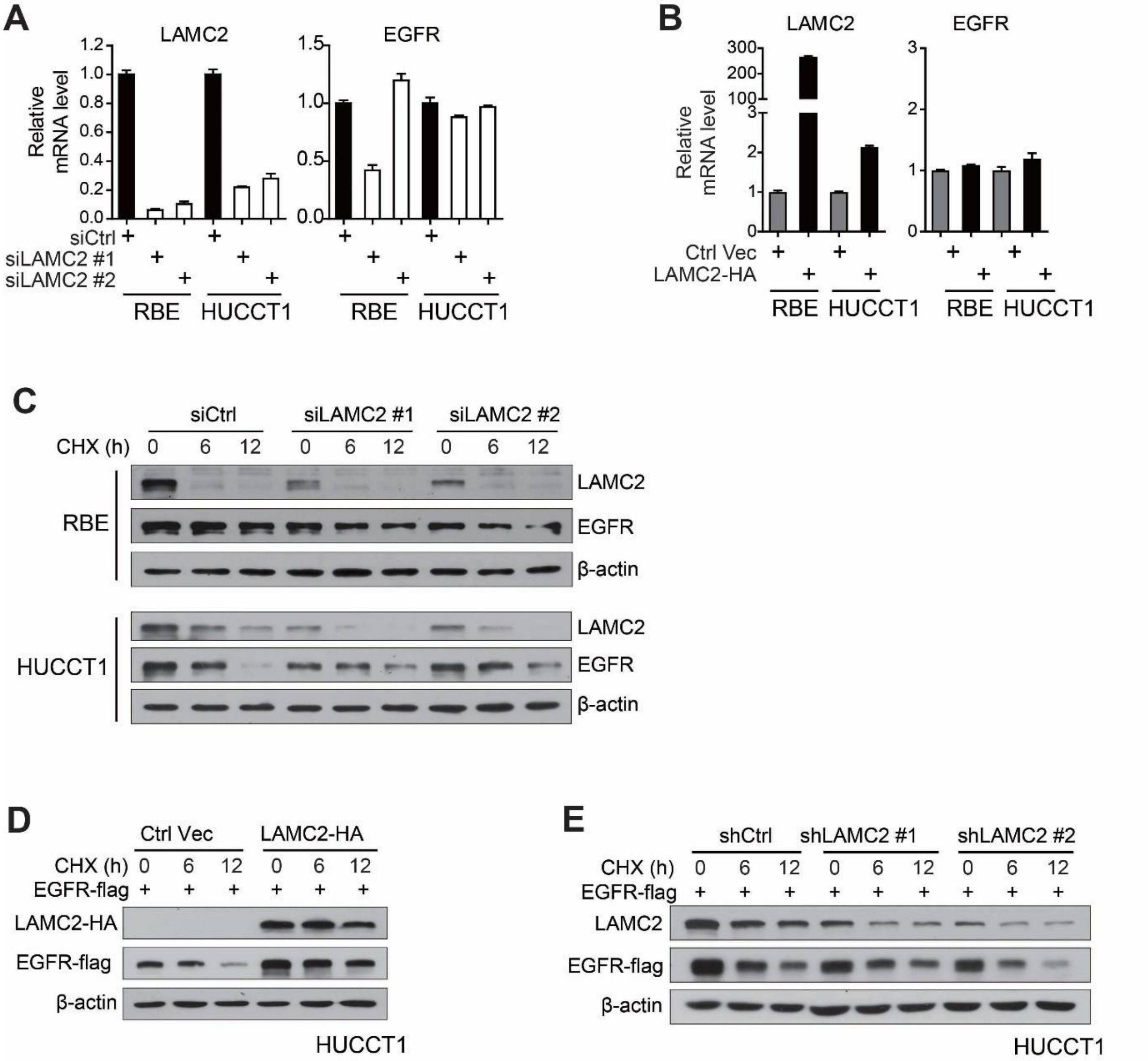

A
LAMC2
EGFR
Relative mRNA level
siCtrl
siLAMC2 #1
siLAMC2 #2
RBE
HUCCT1
B
LAMC2
EGFR
Relative mRNA level
Ctrl Vec
LAMC2-HA
RBE
HUCCT1
C
siCtrl
siLAMC2 #1
siLAMC2 #2
CHX (h)
0
6
12
RBE
HUCCT1
LAMC2
EGFR
β-actin
D
Ctrl Vec
LAMC2-HA
CHX (h)
EGFR-flag
LAMC2-HA
EGFR-flag
β-actin
HUCCT1
E
shCtrl
shLAMC2 #1
shLAMC2 #2
CHX (h)
EGFR-flag
LAMC2
EGFR-flag
β-actin
HUCCT1

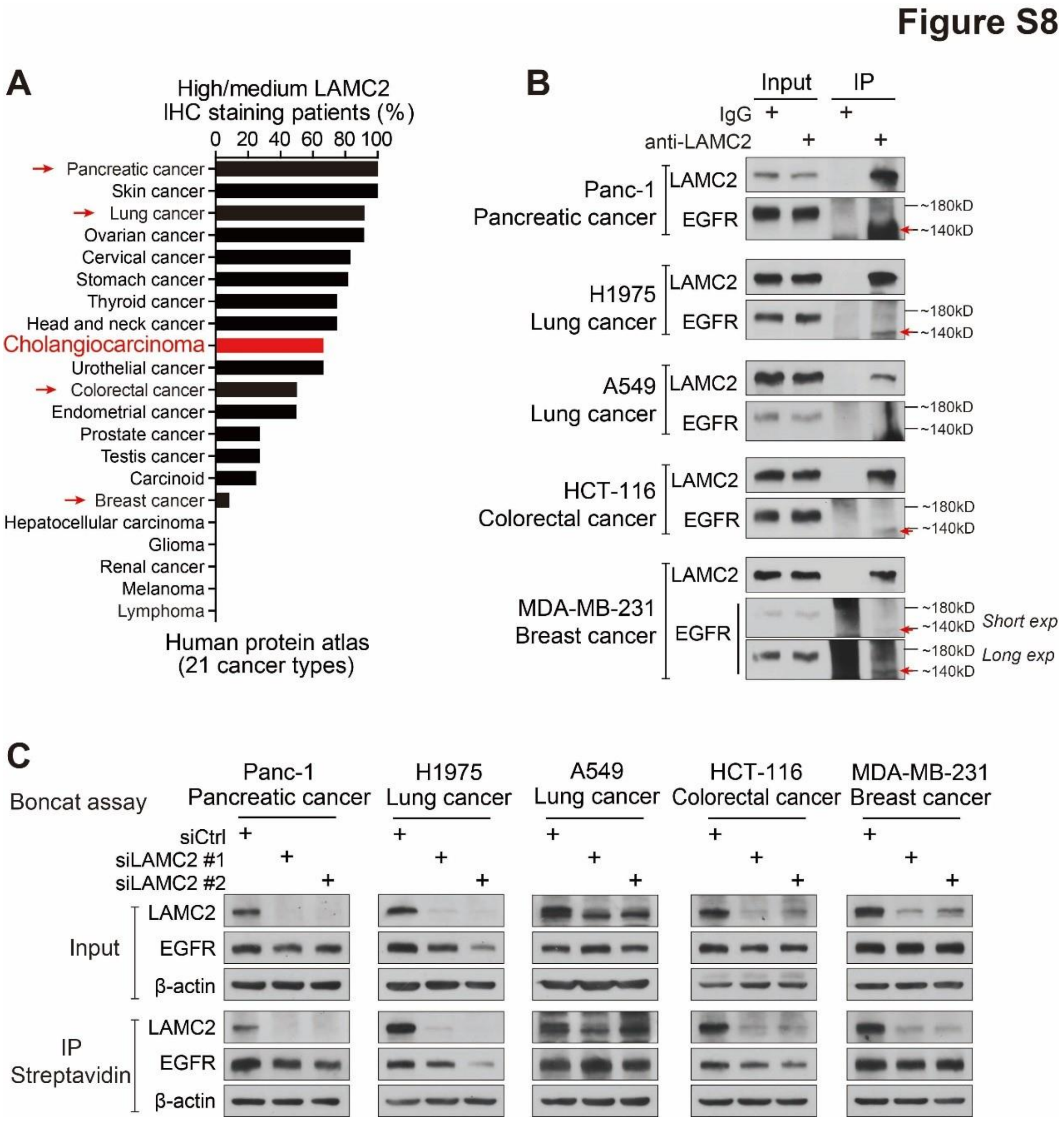
Figure S8
A
High/medium LAMC2 IHC staining patients (%)
0 20 40 60 80 100
Pancreatic cancer
Skin cancer
Lung cancer
Ovarian cancer
Cervical cancer
Stomach cancer
Thyroid cancer
Head and neck cancer
Cholangiocarcinoma
Urothelial cancer
Colorectal cancer
Endometrial cancer
Prostate cancer
Testis cancer
Carcinoid
Breast cancer
Hepatocellular carcinoma
Glioma
Renal cancer
Melanoma
Lymphoma
Human protein atlas (21 cancer types)
B
Input
IP
IgG
anti-LAMC2
Panc-1 Pancreatic cancer
H1975 Lung cancer
A549 Lung cancer
HCT-116 Colorectal cancer
MDA-MB-231 Breast cancer
LAMC2
EGFR
~180kD
~140kD
Short exp
Long exp
C
Boncat assay
Panc-1 Pancreatic cancer
H1975 Lung cancer
A549 Lung cancer
HCT-116 Colorectal cancer
MDA-MB-231 Breast cancer
siCtrl
siLAMC2 #1
siLAMC2 #2
Input
IP Streptavidin
LAMC2
EGFR
β-actin

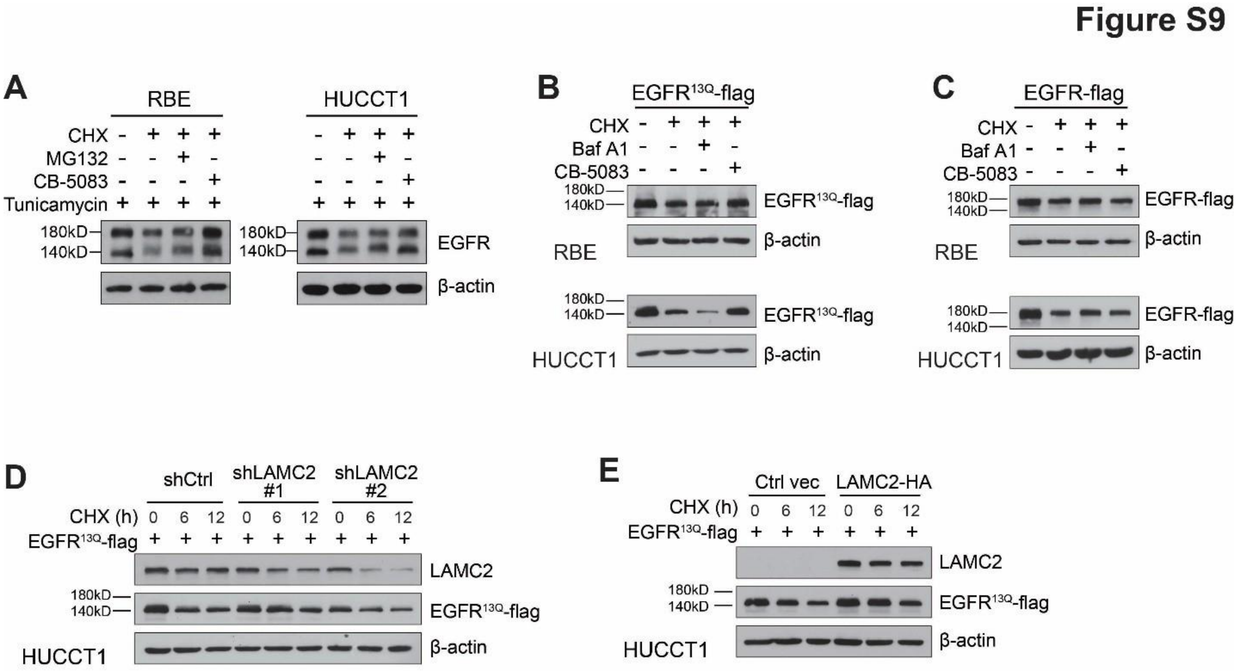
Figure S9
A
RBE
HUCCT1
CHX
MG132
CB-5083
Tunicamycin
180kD
140kD
EGFR
β-actin
B
EGFR13Q-flag
CHX
Baf A1
CB-5083
RBE
HUCCT1
C
EGFR-flag
D
shCtrl
shLAMC2 #1
shLAMC2 #2
CHX (h) 0 6 12
LAMC2
E
Ctrl vec
LAMC2-HA

Figure S10

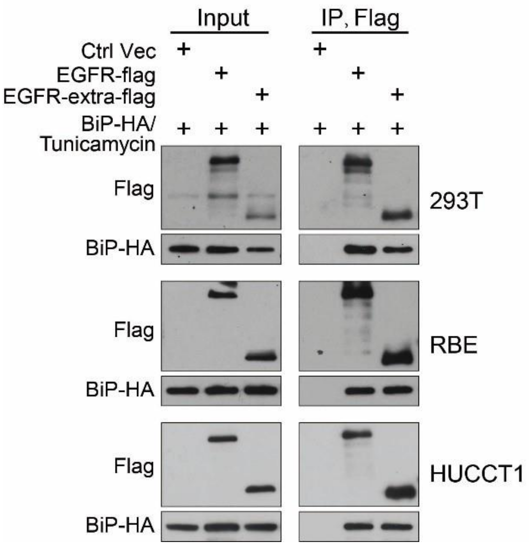

Input
IP, Flag
Ctrl Vec
EGFR-flag
EGFR-extra-flag
BiP-HA/
Tunicamycin
+
Flag
BiP-HA
293T
RBE
HUCCT1

Figure S11

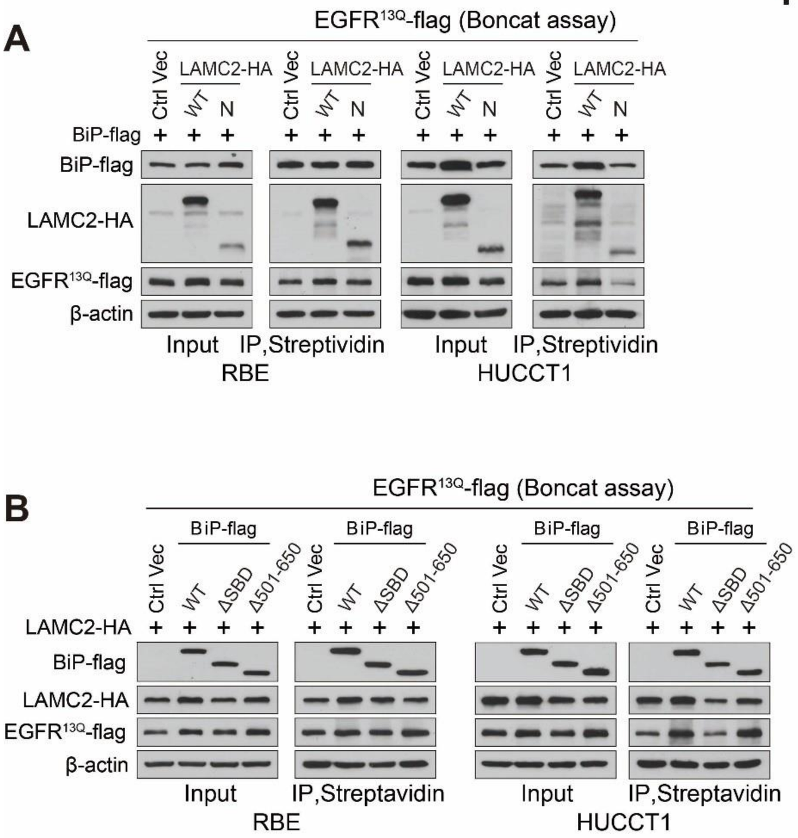

A
EGFR13Q-flag (Boncat assay)
Ctrl Vec
LAMC2-HA
WT
N
BiP-flag
BiP-flag
LAMC2-HA
EGFR13Q-flag
β-actin
Input
IP,Streptividin
RBE
HUCCT1
B
EGFR13Q-flag (Boncat assay)
BiP-flag
Ctrl Vec
WT
ΔSBD
Δ501-650
LAMC2-HA
BiP-flag
LAMC2-HA
EGFR13Q-flag
β-actin
Input
IP,Streptavidin
RBE
HUCCT1

Figure S12

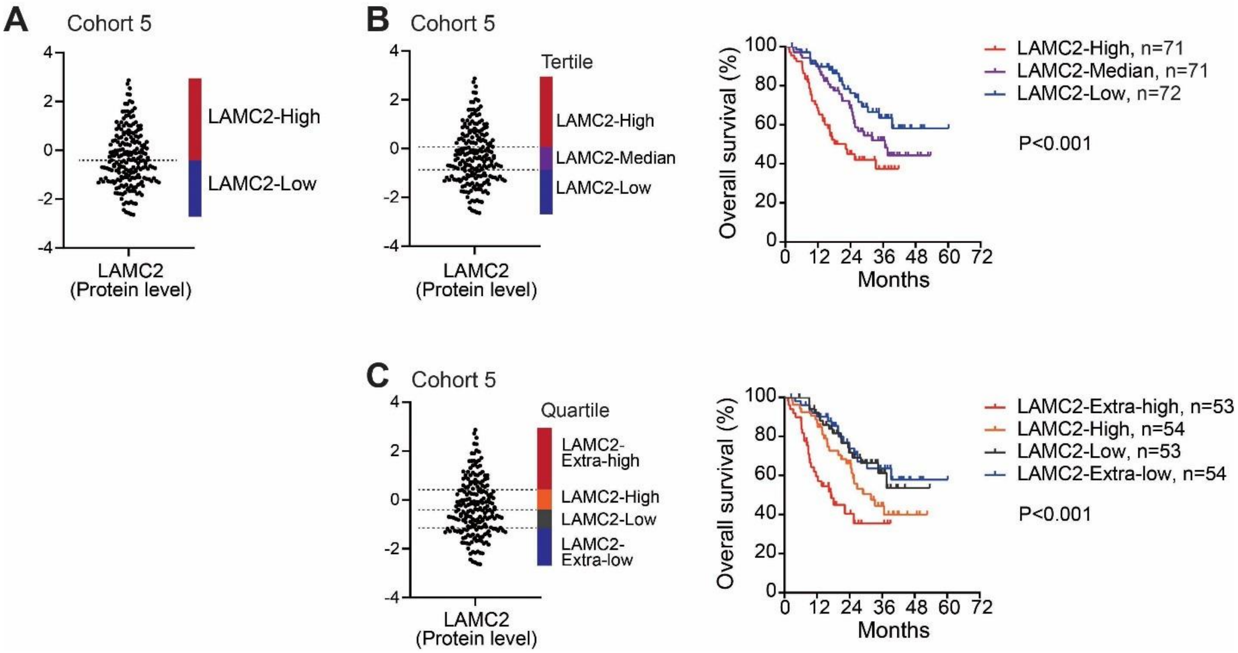
A
Cohort 5
LAMC2-High
LAMC2-Low
LAMC2
(Protein level)
B
Cohort 5
Tertile
LAMC2-High
LAMC2-Median
LAMC2-Low
LAMC2
(Protein level)
Overall survival (%)
Months
LAMC2-High, n=71
LAMC2-Median, n=71
LAMC2-Low, n=72
P<0.001
C
Cohort 5
Quartile
LAMC2-Extra-high
LAMC2-High
LAMC2-Low
LAMC2-Extra-low
LAMC2
(Protein level)
Overall survival (%)
Months
LAMC2-Extra-high, n=53
LAMC2-High, n=54
LAMC2-Low, n=53
LAMC2-Extra-low, n=54
P<0.001

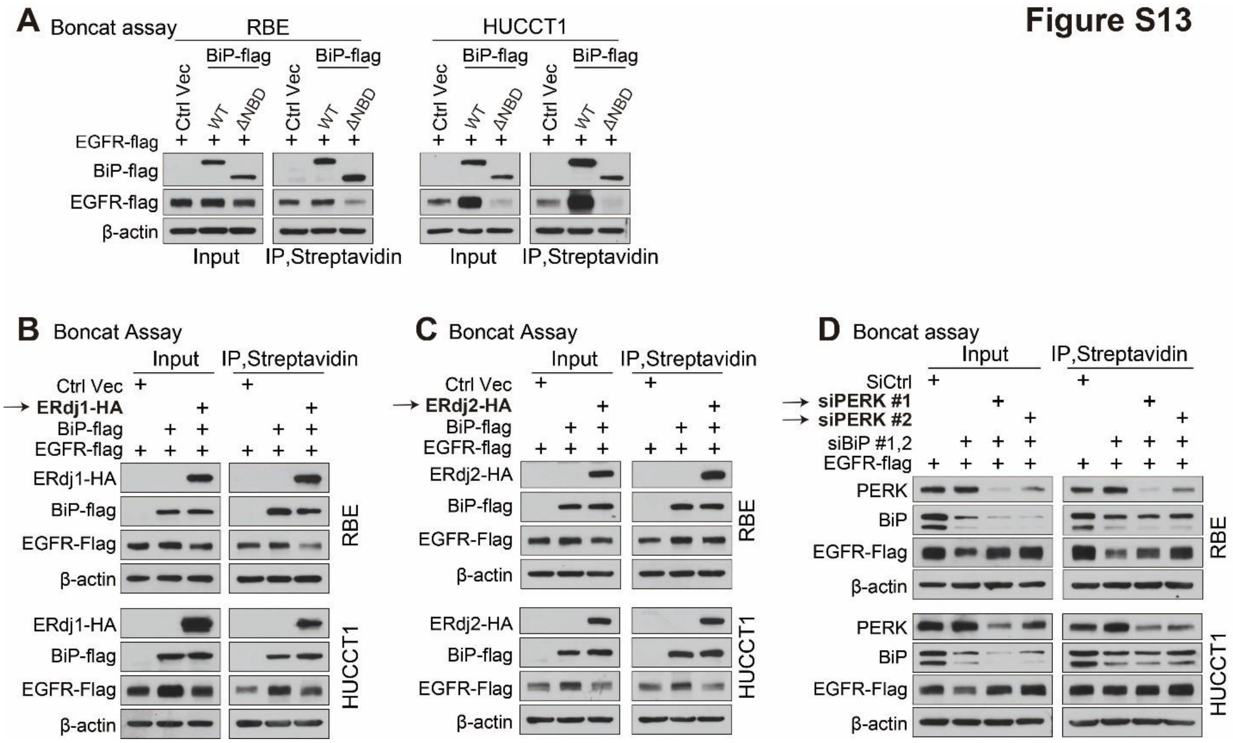
Figure S13
A Boncat assay
RBE
HUCCT1
BiP-flag
Ctrl Vec
WT
ΔNBD
EGFR-flag
BiP-flag
EGFR-flag
β-actin
Input
IP,Streptavidin
B Boncat Assay
Input
IP,Streptavidin
Ctrl Vec
ERdj1-HA
BiP-flag
EGFR-flag
ERdj1-HA
BiP-flag
EGFR-Flag
β-actin
RBE
HUCCT1
C Boncat Assay
Ctrl Vec
ERdj2-HA
BiP-flag
EGFR-flag
ERdj2-HA
BiP-flag
EGFR-Flag
β-actin
D Boncat assay
SiCtrl
siPERK #1
siPERK #2
siBiP #1,2
EGFR-flag
PERK
BiP
EGFR-Flag
β-actin

Figure S14

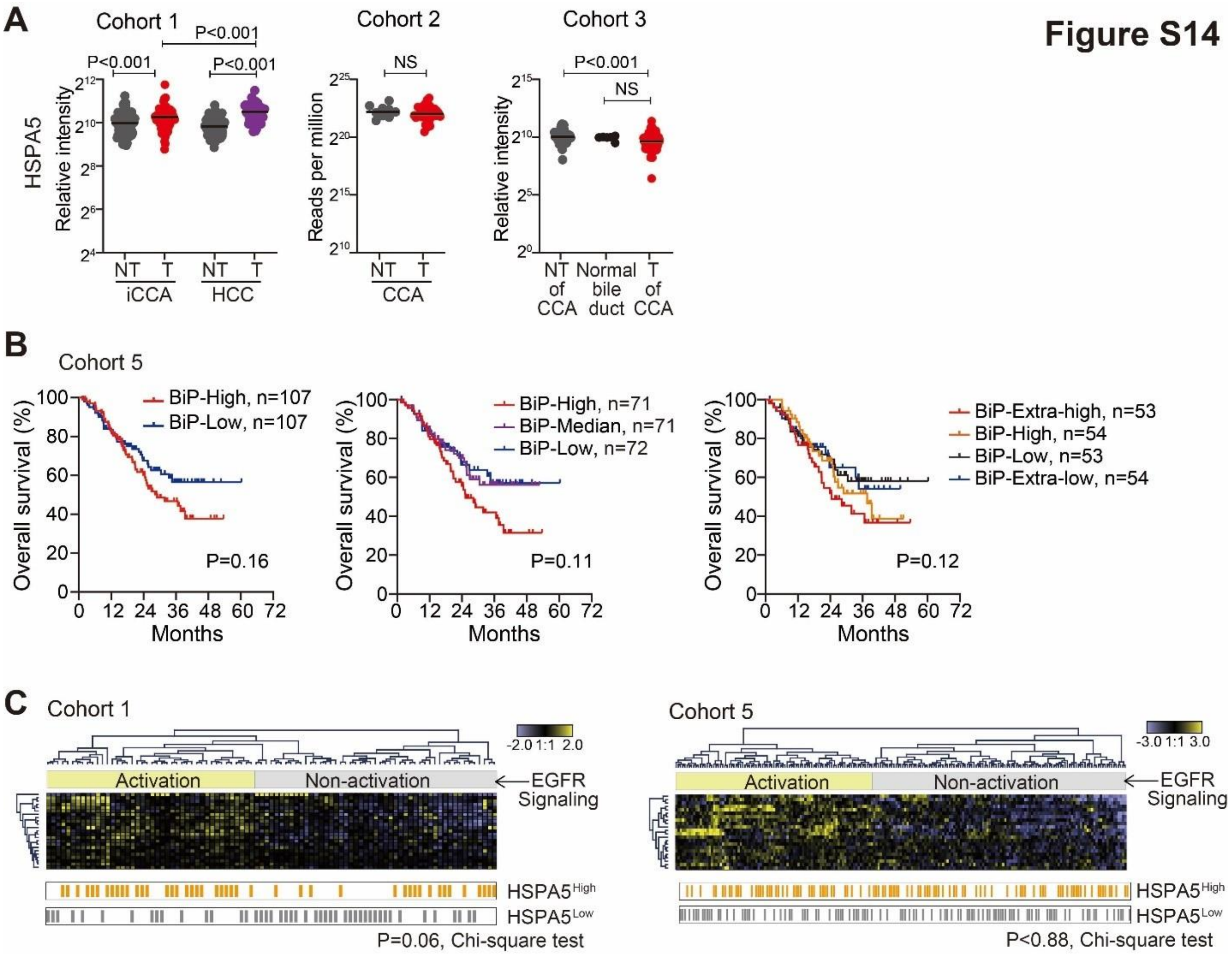
A
Cohort 1
P<0.001
P<0.001
P<0.001
HSPA5
Relative intensity
NT T NT T
iCCA HCC
Cohort 2
NS
Reads per million
NT T
CCA
Cohort 3
P<0.001
NS
Relative intensity
NT of CCA
Normal bile duct
T of CCA
B
Cohort 5
BiP-High, n=107
BiP-Low, n=107
P=0.16
BiP-High, n=71
BiP-Median, n=71
BiP-Low, n=72
P=0.11
BiP-Extra-high, n=53
BiP-High, n=54
BiP-Low, n=53
BiP-Extra-low, n=54
P=0.12
Overall survival (%)
Months
C
Cohort 1
-2.0 1:1 2.0
Activation
Non-activation
EGFR Signaling
HSPA5High
HSPA5Low
P=0.06, Chi-square test
Cohort 5
-3.0 1:1 3.0
Activation
Non-activation
EGFR Signaling
HSPA5High
HSPA5Low
P<0.88, Chi-square test
